# Integrated optimization of energy storage and green hydrogen systems for resilient and sustainable future power grids

**DOI:** 10.1038/s41598-025-09408-x

**Published:** 2025-07-15

**Authors:** Ahmed M. Asim, Ahmed S. A. Awad, Mahmoud A. Attia

**Affiliations:** 1 Department of Electrical Power and Machines Engineering, Faculty of Engineering, Ain Shams University, Cairo, Egypt; 2 Department of Electrical and Computer Engineering, University of Windsor, Windsor, ON N9B 3P4 Canada

**Keywords:** Multi-objective optimization, Energy storage systems (ESS), Renewable energy integration, Green hydrogen production, Oman vision, Pumped hydro storage (PHES), Compressed air energy storage (CAES), Energy grids and networks, Electrical and electronic engineering, Energy storage, Batteries, Hydrogen storage

## Abstract

This study presents a novel multi-objective optimization framework supporting nations sustainability 2030–2040 visions by enhancing renewable energy integration, green hydrogen production, and emission reduction. The framework evaluates a range of energy storage technologies, including battery, pumped hydro, compressed air energy storage, and hybrid configurations, under realistic system constraints using the IEEE 9-bus test system. Results show that without storage, renewable penetration is limited to 28.65% with 1538 tCO_2_/day emissions, whereas integrating pumped hydro with battery (PHB) enables 40% penetration, cuts emissions by 40.5%, and reduces total system cost to 570 k$/day (84% of the baseline cost). The framework’s scalability is confirmed via simulations on IEEE 30-, 39-, 57-, and 118-bus systems, with execution times ranging from 118.8 to 561.5 s using the HiGHS solver on a constrained Google Colab environment. These findings highlight PHB as the most cost-effective and sustainable storage solution for large-scale renewable integration.

## Introduction

Different Visions 2030–2040 mark a transformative shift from hydrocarbon reliance towards a diversified, sustainable energy future, driven by the need for enhanced energy security and economic sustainability. For instance, Oman vison defined an ambitious target: reducing the oil sector’s GDP contribution from 39% (2017) to 8.4% by 2040. This also aims to increase renewable energy consumption to 35–39% of total energy use, with an interim goal of 20% by 2030^1^. A critical component is developing robust solar and wind infrastructure and significantly scaling clean hydrogen production from 32,500 metric tons to 1 million annually by 2030, and further to 8.5 million by 2050^2^. This entails a complete reimagining of Oman’s energy infrastructure, including smart grids, energy storage, and modern distribution networks with a strong commitment to environmental protection and green technologies, positioning Oman as a regional leader in sustainable energy^1^.

To achieve these goals, the Oman Electricity Transmission Company (OETC) is actively integrating large-scale renewable projects, such as the 500 MW Ibri II Solar IPP, with further solar expansions in Manah (1000 MW), Ibri III (500 MW), and A Kamil (500 MW), alongside wind projects in JBB (100 MW), Duqm (200 MW), Ras Madrakah (200 MW), and Dhofar II (100 MW) planned for connection to the Main Interconnected System (MIS) by 2030^3^. While this significant renewable penetration supports sustainability objectives, it introduces substantial technical challenges. Primary concerns include the inherent intermittency of solar and wind power generation and the reduction in system inertia due to the displacement of conventional synchronous generators, both of which critically affect grid stability and operational reliability^3^.

Energy Storage Systems (ESSs) present crucial opportunities to address these challenges, enhancing renewable energy integration in Oman, lowering operational costs, and reducing fossil fuel consumption by managing intermittency and stabilizing the grid^4,5^. Current research highlights various ESS technologies. These technologies vary in their applicability: lithium-ion batteries offer high efficiency but face scalability and environmental issues; Pumped Hydro Energy Storage (PHES) provides large-scale storage but is geographically limited; Compressed Air Energy Storage (CAES) also presents a viable option for large-capacity, long-duration storage, contingent on geographical suitability; flywheels excel in rapid response but lack long-term capacity; and thermal storage suits specific applications^6^. Emerging innovations include solid-state batteries, hydrogen for long-duration storage, and Artificial Intelligence/Machine Learning techniques for optimized management^6^.

### Literature review

Previous studies have explored optimal sizing and location of ESS with different objectives.

Authors of^7^ introduced an advanced distributed planning framework for integrated electricity and natural gas systems (IEGS), explicitly incorporating Regional Integrated Energy Systems (RIESs) and addressing the realistic scenario of multi-stakeholder ownership across gas networks (GN), electricity networks (EN), and RIESs. Departing from conventional centralized joint planning approaches, the proposed model leveraged the Alternating Direction Method of Multipliers (ADMM) to enable decentralized coordination, allowing each agent to independently optimize their objectives while ensuring convergence on shared operational variables such as transmission flows. Validated through a case study using the IEEE 24-bus and Belgian 20-node systems, the model demonstrated improved adaptability and responsiveness to localized variations, along with effective negotiation-based planning among stakeholders. Sensitivity analyses further assessed the influence of penalty parameters and convergence thresholds on algorithm performance. However, the model faced several limitations, including convergence challenges due to the non-convexity of mixed-integer programming (MIP), high computational burden compared to centralized models, and an observed increase of 4.86% in overall planning costs due to distributed inefficiencies. Additionally, the framework relied on simplified assumptions, such as static market conditions and uniformly cooperative stakeholder behavior, which may not fully capture the complexity of competitive or deregulated energy environments. These limitations suggested the need for future research to enhance convergence robustness, integrate economic uncertainty, and extended the model’s applicability to larger and more dynamic multi-energy systems. Authors of^8^ presented a comprehensive framework for multi-objective optimization of an interactive buildings-vehicles energy sharing network that leveraged grid-responsive strategies, diverse renewable integrations, and hybrid storage systems. By employing the advanced Pareto archive NSGA-II algorithm, the study effectively minimized equivalent CO_2_ emissions and import costs while maximizing energy flexibility. The proposed system achieved notable performance improvements over conventional isolated configurations, including a 7.5% reduction in emissions (from 147.4 to 136.4 kg/m^2^·a), an 8.5% decrease in grid import costs (from 212.7 to 194.6 HK$/m^2^·a), and substantial enhancements in energy flexibility metrics, such as shifting 52.48% of surplus renewable energy compared to 33.6% in the baseline scenario. A key innovation was the integration of quantifiable flexibility indicators like RSR and GSR, offering valuable insight into system responsiveness and storage effectiveness under dynamic conditions. Nevertheless, limitations remained in the deterministic modeling of vehicle behavior, the omission of battery production and degradation impacts, and a lack of in-depth economic feasibility analysis regarding capital investment and ROI.

Complementing this work, authors of^9^ offered a multidisciplinary perspective on urban energy systems by emphasizing the cross-sectoral integration of buildings, transportation, and power grids to support the development of sustainable and climate-resilient smart cities. The study underscored the importance of technologies such as bidirectional EV charging, hydrogen refueling stations, and building prosumers in enhancing energy efficiency and system resilience. By incorporating climate modeling, artificial intelligence, energy resilience indicators, and techno-economic-environmental evaluations, the authors proposed a holistic roadmap for city-scale planning and energy management. However, this analysis was primarily conceptual and lacked empirical validation, detailed methodological descriptions of optimization or AI frameworks, and region-specific economic assessments. Furthermore, while societal and regulatory barriers were acknowledged, they were not explored in sufficient depth.

In^10^, another study advanced a co-simulation framework combining MATLAB and TRNSYS to enable multi-objective optimization and component sizing of a solid oxide fuel cell-based combined cooling, heating, and power (SOFC-CCHP) system for green buildings. Utilizing a semi-empirical surrogate model of the SOFC, the study optimized the battery, electrolyzer, and SOFC subsystems to simultaneously enhance energy efficiency and reduce annual costs while accounting for performance degradation. Results indicated that increasing the size of the electrolyzer and SOFC improved energy efficiency by 13.64% and 2.19%, respectively, with annual costs ranging between $67,230 and $73,250. A novel application of ANOVA and Pareto-based decision-making added robustness to the design process. Despite its methodological strengths, the study relied on idealized gas assumptions, excluded water crossover effects, and did not account for uncertainties in input data or operational variability, limiting its applicability in real-world dynamic environments.

In a related context, authors of^11^ conducted an in-depth study on sustainable interactive energy sharing districts that incorporated electrochemical battery storage. This work explored multi-directional energy exchanges among PV systems, wind turbines, buildings, EVs, and microgrids, and integrated both semi-empirical and machine learning models for battery lifetime estimation. The study proposed deterministic and stochastic control frameworks, emphasizing stakeholder-based optimization approaches and employing multi-criteria decision-making tools such as Shannon entropy, Euclidean distance, and fuzzy logic. It provided valuable insights into system design, battery sizing, carbon reduction, and cost-effectiveness, with a strong focus on improving energy flexibility and resilience. Nonetheless, the research was largely simulation-based and lacked empirical validation. Moreover, while battery degradation was thoroughly addressed, economic trade-offs and real-time data limitations in machine learning applications were not fully examined.

Building upon the theme of battery longevity, another study^12^ proposed a hierarchical control strategy that integrated thermal and electrical storage to reduce battery cycling ageing and improve energy flexibility. By applying depth-of-discharge (DoD)-based control and utilizing surplus renewable energy for recharging thermal systems such as air conditioning and hot water tanks, the strategy significantly mitigated battery degradation and extended system life. Results demonstrated an increase in relative battery capacity from 94.21 to 95.46% annually and system cost reductions of up to 19.33% for lead-acid batteries. However, this work remained theoretical, lacking real-world implementation or sensitivity analyses that would consider market conditions, infrastructure constraints, or policy incentives.

In^13^, a climate-adaptive design framework for Zero-Energy Buildings (ZEBs) was introduced, proposing an integrative "kWp-kWh-m^2^" approach that coupled PV generation with optimally sized battery storage across various Chinese climate zones. The study employed a life-cycle analysis model incorporating economic, environmental, and policy parameters under different Representative Concentration Pathway (RCP) scenarios. It revealed that battery integration significantly improved renewable penetration, with decarbonization potential reaching up to 70%, especially in centralized systems. The authors provided strategic battery sizing recommendations—ranging from 3.75–4 kWh/kWp for centralized systems to 1.75–2.75 kWh/kWp for distributed setups, while highlighting the influence of electricity pricing, carbon intensity, and climate variations. Despite its comprehensive modeling and policy relevance, the study lacked empirical case studies and broader regional comparisons, which limited its global generalizability.

Furthering the agenda for sustainable electrification, authors of^14^ proposed a lifecycle carbon neutrality strategy grounded in an electricity-driven circular economy. The study explored five scenarios integrating renewable energy, vehicle-to-everything (V2X) interaction, and EV battery cascade utilization. Among these, the synergistic scenario combining V2X and second-life battery reuse proved most effective, achieving negative carbon intensity and strong economic viability across varied climate zones. Moreover, the analysis extended globally, supported by policy instruments such as production and investment tax credits. However, the study did not fully consider the transitional dynamics of power grid decarbonization or the integration of large-scale centralized renewables like offshore wind, suggesting the need for further research into broader energy contexts.

In^15^, a novel lifecycle design methodology for renewable-battery-consumer energy systems was introduced, focusing on battery capacity optimization through the "M-value" (matching degree) method. This approach offered a significant improvement over the conventional "U-value" (uniformity) method by aligning PV generation more effectively with building energy demand. The framework accounted for performance degradation, carbon emissions, and economic outcomes, and established a scalable area-kWp-kWh design guideline. Simulation results across different building types and climate zones in China demonstrated lower lifecycle carbon intensity and improved net present value (NPV). Nevertheless, the scope was limited to solar PV and Li-ion batteries, excluding other renewable technologies and the potential contributions of electric vehicles to energy balancing.

Other researchers addressed the optimal sizing and location of photovoltaic generation systems (PVGS) and battery energy storage systems (BESS) to enhance power loss reduction, voltage profile improvement, and voltage unbalance in an unbalanced distribution system. It employs a refined genetic algorithm for optimization, considering investment costs and operational constraints. Other researchers presented a novel optimization framework for the optimal siting and sizing of distributed renewable generation and energy storage systems. It utilized four distinct load models and three metaheuristic approaches, with the Elephant Herding Optimization (EHO) emerging as the best performer for voltage stability and real power loss reduction. The findings suggest that the Modified Ant Lion Optimization (ALO) is most effective under ideal conditions, particularly when wind and irradiance percentages are 60% or greater^16,17^. While authors of^18^ presented a deterministic planning model for the optimal sizing and location of battery energy storage systems (BESS) to support renewable energy sources. It utilized a mixed-integer nonlinear programming approach to minimize investment and operational costs while satisfying system constraints. The model was tested on a modified IEEE 14-bus system, indicating its applicability for future scenarios. In^19^, researchers discussed a method for optimal sitting and sizing energy storage (ES) systems to support renewable energy integration, focusing on minimizing the expected operating and investment costs. It analyzes various parameters, including the maximum number of storage locations and renewable generation capacity. It was evaluated using a realistic model of the Western Electricity Coordinating Council (WECC) interconnection, consisting of 240 buses and 448 lines. While in^20^, Authors established an optimization method for determining the optimal sizing and location of energy storage plants to support renewable energy. This approach involved pre-selecting locations based on load, renewable energy capacity, and distances to neighboring nodes. The multi-objective artificial bee colony algorithm was employed to maximize peak-shaving profit, minimize investment costs, and reduce active network loss. This approach effectively enhanced renewable energy utilization by optimizing both the location and capacity of energy storage facilities. Authors of^21^ focused on determining the optimal capacity and location of energy storage systems (ESS) to support grid stability in the presence of renewable energy sources (RESs). It emphasized that ESS can provide virtual inertia, mitigating frequency fluctuations caused by the low inertial characteristics of RESs. The developed model considered power grid constraints, including voltage, angle, and line capacity limits, and was evaluated using the New England IEEE 39-bus system to ensure cost-effectiveness and stability. While in^22^, researchers found that Energy storage systems can significantly improve frequency response in power systems with high renewable penetration, particularly in scenarios with low inertia due to synchronous generator displacement. Also found that the optimal placement of storage could mitigate frequency disturbances, enhancing overall system reliability during transients. Authors of^23^ deduced that Storage technologies could absorb surplus renewable energy, reducing the need for additional capacity by 24–44% under favorable economic conditions. While authors of^24^ discussed that the integration of storage not only lowers greenhouse gas emissions but also decreases overall energy costs by optimizing the use of available renewable resources. They deduced that geographic and seasonal variations in renewable energy availability necessitate tailored storage solutions to meet local demand effectively. In^25^, researchers concluded that although the benefits of energy storage in supporting renewable energy integration are evident, significant challenges persist, particularly the high costs of certain storage technologies and the need for supportive policies to enable widespread adoption.

### Hydrogen literature

Green hydrogen, produced through water electrolysis using renewable energy, plays a vital role in decarbonizing energy and transport sectors by enabling flexible integration of intermittent renewables. Hydrogen fuel cell vehicles (HFCVs) are central to this integration, supported by technological advances and expanding refueling infrastructure. Despite this progress, significant technological, economic, and regulatory barriers hinder the growth of the hydrogen economy. This review highlights current achievements and ongoing challenges in deploying HFCVs and scaling green hydrogen production, and proposes a roadmap for future research, policy, and investment to advance hydrogen as a key component of sustainable energy and transport systems^26^. This section presents a brief critical review of hydrogen optimization with renewable energy integration, including comments on the limitations of existing studies.

Authors of^27^ developed an integrated renewable energy–refinery hydrogen management system that combined energy storage and direct utilization to enhance hydrogen utilization efficiency in refinery operations. They introduced a novel superstructure that encompassed green hydrogen production via water electrolysis and hydrogen compression powered by wind energy, supported by underground hydrogen storage (UHS) units. A nonconvex MINLP model was formulated to optimize the system’s design, incorporating mass and energy balances, compressor configurations, and cost functions. Through a hierarchical algorithm, the study demonstrated that the proposed approach reduced total annual costs by 21.21% under abandoned wind pricing and 13.49% under normal pricing, with further reductions projected for 2035 and 2050. It was also found that using wind power for hydrogen compression yielded more favorable economics than standalone green hydrogen production, especially as wind electricity costs decreased. Overall, the study emphasized the feasibility of directly integrating fluctuating renewable energy into refinery hydrogen systems while minimizing energy conversion losses. Despite its innovative contributions, the research relied on idealized assumptions regarding system behavior, constant hydrogen purity, and geographic availability of UHS units. Moreover, the lack of empirical validation and sensitivity analysis under broader economic and policy uncertainties limited the real-world applicability of the proposed model.

In^28^, authors conducted a comprehensive methodological review of renewable hydrogen system modeling and optimization, with a particular focus on integrating machine learning to enhance system performance and decision-making. The study critically evaluated limitations in traditional modeling approaches and highlighted the need for dynamic representations of solar PV, electrolyzer, and fuel cell performance. It demonstrated that machine learning techniques—such as neural networks and support vector machines—significantly improved forecasting accuracy, component behavior modeling, and load profile development, particularly in data-scarce regions like the Global South. Additionally, the authors proposed a novel metric—Levelized Value Addition (LVA)—to incorporate socio-economic considerations into hydrogen system evaluations. An integrated, evidence-based multi-criteria decision-making (MCDM) framework combining both multi-objective and multi-attribute methods was also introduced. By identifying best practices in optimization, load profile creation, and economic assumption modeling, the review offered a structured pathway toward more realistic, data-driven, and socially inclusive system designs. However, the work remained largely conceptual, lacking empirical case studies to validate the proposed frameworks. Furthermore, although machine learning integration was extensively discussed, practical challenges such as data scarcity, interpretability, and computational complexity were not fully resolved. The application of socio-economic metrics like LVA also lacked real-world demonstrations across different contexts, limiting their immediate implementation potential.

Similarly, authors of^29^ proposed a two-stage distributed robust optimization model for scheduling a hydrogen production system based on renewable energy sources (H2-RES), taking into account the uncertainties in solar and wind power generation as well as the flexibility of electric loads. The first stage optimized the capacity configuration of key components—electrolyzers, hydrogen compressors, and hydrogen storage tanks, with the aim of minimizing investment costs. The second stage focused on dynamic scheduling of system operation under worst-case scenarios using the Column and Constraint Generation (C&CG) algorithm to minimize operating costs. The model incorporated flexible load modeling, including transferable and reducible loads, and was applied to a real hydrogen project in Zhangjiakou, where it demonstrated improved economic performance and better alignment with actual system configurations. The study effectively highlighted the importance of flexible demand response and robust planning in enhancing the reliability and cost-effectiveness of hydrogen systems integrated with renewables. Nevertheless, it was constrained by its heavy reliance on simulation and the absence of operational data for validation. Behavioral and regulatory factors influencing real-world user participation in flexible load schemes were not deeply addressed, and the worst-case focus of the uncertainty treatment potentially oversimplified the stochastic nature of renewable fluctuations.

In a broader system context, authors of^30^ developed a comprehensive modeling and optimization framework for a large-scale renewables-based hydrogen system that encompassed the entire production–storage–transportation–utilization chain (PSTUH_2_S). The study considered multiple hydrogen production sources—renewables, fossil fuels, and grid power—as well as various storage options, transport methods, and sectoral demands spanning industry, power, construction, transport, and aerospace. A multi-objective nonlinear optimization model was formulated to simultaneously maximize economic benefit, increase renewable energy consumption, and minimize carbon emissions. Solved using a hybrid approach combining nonlinear programming, the CPLEX solver, and piecewise time series simulation, the model was applied in northwest China for the period 2025–2035. The results demonstrated significant economic and environmental gains, including cost savings up to USD 865.87 million and reductions in renewable energy curtailment and emissions. Sectoral analysis revealed industrial hydrogen as the dominant demand, though rapid growth potential was observed in power and transportation sectors. Despite its extensive modeling, the study depended on deterministic forecasts and assumed fixed operational parameters—such as hydrogen blending ratios and ideal electrolyzer behavior—potentially limiting its responsiveness to real-world variability. Additionally, socio-institutional considerations such as regulatory frameworks, market dynamics, and infrastructure readiness were not sufficiently explored.

Lastly, authors of^31^ conducted a detailed parametric and optimization study on solar/wind-based hybrid renewable hydrogen production in Kuqa, China. They explored six scenarios under both grid-connected and off-grid configurations using HOMER and TRNSYS simulations to evaluate performance under varying energy conditions. The PV/WT (G5) system was identified as the most effective grid-connected configuration, while the PV/WT/FC (S6) system was optimal for off-grid applications. Both systems demonstrated strong technical and economic performance, with low energy costs, reduced capacity shortages, and improved hydrogen utilization. The study further optimized system configurations by refining load profiles and adjusting component sizes, leading to reduced grid dependency in G5 and lower excess energy rates in S6. These findings underscored the value of careful component-load matching to enhance system efficiency in arid, resource-abundant settings. However, the research remained confined to simulation and lacked empirical testing. Operational assumptions, such as consistent component behavior and static user demands—may not hold in real-world deployments. Furthermore, the study’s narrow focus on techno-economic metrics excluded policy, regulatory, and environmental dimensions necessary for scalable and sustainable deployment.

### Research gap

While existing literature provides valuable insights into the optimal sizing and siting of energy storage systems (ESS) to support renewable energy integration, several limitations persist that highlight the need for further research. Many studies rely on deterministic or idealized scenarios, often neglecting the impact of uncertainties in renewable generation, demand variability, and market conditions. Additionally, most models assume static network conditions and do not fully capture the dynamic behavior of power systems under high renewable penetration. The optimization methods employed, though diverse, are often tested on small or simplified test systems, limiting their scalability and applicability to real-world, large-scale grids. Furthermore, several approaches overlook the coordination among multiple stakeholders, regulatory constraints, and economic incentives required for practical implementation. High investment costs and lack of detailed techno-economic analysis for different storage technologies also remain underexplored. These gaps underscore the need for more comprehensive, robust, and scalable models that incorporate uncertainty, system dynamics, and policy frameworks to support effective ESS planning in future power systems.

This paper presents a novel multi-objective optimization framework designed specifically to support Oman’s Vision 2040 energy transition goals. The study systematically evaluates how various energy storage systems (ESS), including pumped hydro storage, compressed air energy storage, batteries, and hybrid configurations, perform across different renewable energy investment scenarios. The framework simultaneously optimizes three critical objectives: maximizing renewable energy integration, minimizing carbon emissions, and enabling green hydrogen production from surplus energy.

Unlike previous approaches, the model incorporates realistic storage charge/discharge efficiency losses and explicitly accounts for transmission line flow constraints as a proxy for N-1 security requirements. This allows for determination of maximum renewable penetration levels that maintain system reliability while identifying the most suitable storage technologies for Oman’s specific needs. Moreover, this study includes a detailed techno-economic assessment of each storage configuration, which is often overlooked in the literature. By validating our framework on the IEEE 9-bus test system, we demonstrate its robustness and applicability, offering practical insights for grid planners and policymakers. Furthermore, the scalability of the proposed optimization framework was evaluated using IEEE 30, IEEE 39, IEEE 57, and IEEE 118-bus systems, demonstrating its computational efficiency and practical applicability on larger grid models.

The rest of this paper is organized as follows: The paper’s contributions are provided in Section “Contributions”. The model formulation is described in Section “Model formulation”. The results and discussion are covered in Section “Results and discussion”. The Scalability is proved in Section “Computational scalability”. Finally, Section “Conclusion” includes conclusions and suggested future studies.

## Contributions

This study proposes an optimization framework to support Oman’s Energy Transition 2040 objectives by maximizing Renewable Energy (RE) integration, facilitating electrolyzer-based green hydrogen production, and minimizing carbon emissions while maintaining strict compliance with power system operational constraints^1^. The developed framework is validated through a multi-stage scenario analysis on the IEEE 9-bus benchmark power system, structured as follows:

1. *Baseline Emissions Assessment*: Quantification of grid emissions under conventional generation without renewable energy penetration.
2. *Maximum Renewable Energy (RE) Integration*: Determining the highest achievable level of renewable energy penetration—expressed as the percentage ratio of electrical energy supplied from renewable sources to the total electrical energy supplied to the system—while maintaining system security constraints.
3. *Enhanced Renewable Energy Utilization*: Increasing the percentage of renewable energy effectively used, whether as electricity or converted into green hydrogen. This includes absorbing surplus renewable generation by incorporating green hydrogen production capabilities into the system.
4. *Improving RE Penetration Levels*: Using various types of storage mix to achieve penetration levels near 40%. Storage configurations:
  - Battery
  - Pumped hydro
  - Compressed Air Energy Storage (CAES)
  - Pumped hydro with battery
  - Compressed Air Energy Storage with battery
5. *Cost analysis*: Introduces a comprehensive daily cost framework combining operational generation costs, social carbon credit revenues, and the equivalent daily cost of storage based on Levelized Cost of Storage (LCOS). This approach captures real-time operational expenses, monetizes CO_2_ emissions reductions, and accounts for the full lifecycle cost of storage technologies. It enables a realistic and concise economic evaluation of renewable integration and storage scenarios, supporting informed investment and policy decisions.
6. *Scalability*: Proved computational scalability through IEEE 30, IEEE 39, IEEE 57 and IEEE 118 bus systems with a timeframe of 1 year.

Finally, a detailed comparative study including techno-economic assessment is conducted showing the superiority of pumped hydro scenarios.

## Model formulation

This section presents the detailed mathematical structure of the large-scale power system optimization model. It defines the fundamental sets, indices, decision variables, and parameters used to represent the system components and their behavior over a specified time horizon. The formulation encompasses the objective function, which maximizes renewable penetration level, and a comprehensive set of constraints that supports various types of storage, operational limits, energy balance, and policy targets (such as emissions caps and hydrogen production requirements). The model is formulated as a linear program, leveraging the DC power flow approximation for the electrical network to ensure scalability to real power systems as Oman’s network. The model is implemented using PyPSA (Python for Power System Analysis), an open-source Python package designed to simulate and optimize modern energy systems across multiple time steps, with model execution performed on the Google Colab platform^32,33^.

### Indices

The indices defined below are used throughout the model to represent the various components and time steps in the system, providing a structured framework for the mathematical formulation:

- $t\in T$: Set of hourly time steps (snapshots),$T=\left\{\text{1,2},\dots ,24\right\}$
- $n\in N$: Set of buses (AC bus, DC bus, Hydrogen bus, … etc.) in power network.
- $g\in G$: Set of generators (conventional or renewable).
- $l\in L$: Set of transmission lines. For a line $l$ connecting bus $a$ to bus $b$, we may denote it as $l\left(a,b\right)$.
- $s\in S$: Set of Storage units (e.g., Battery).
- $es\in ES$: Set of energy stores (e.g., hydrogen store, heat store). Typically connected to non-AC buses or via energy links.
- $el\in EL$: Set of energy links (e.g., hydrogen electrolyzer, DC link, thermal link). For a link $el$ connecting bus $a$ to bus $b$, we may denote it as $el\left(a,b\right)$.
- $m\in M$: Set of meshes or cycles in the network topology.

### Objective

The objective of the optimization model is to balance multiple goals simultaneously: maximizing the integration of renewable energy sources, minimizing carbon emissions from conventional generators, and ensuring efficient operation of the power system. To achieve this, a weighted sum approach is employed, where each generator’s output is assigned a preference weight. Renewable generators are given negative weights to encourage their utilization, while conventional generators receive positive weights, with higher values for those with greater emission intensities. This formulation allows the model to prioritize cleaner energy sources while maintaining system reliability. The objective function is formulated as follows:

1 $$\text{min}{\sum }_{n,g,t}{W}_{n,g}\cdot {p}_{n,g,t}$$

${W}_{n,g}$: Preference weights assigned to generator $g$ at bus $n$. Unlike traditional cost-based coefficients, these weights implement a multi-objective strategy:

${p}_{n,g,t}$: Active power output of generator $g$ at bus $n$ at time $t$ (MW).

- Negative weights for renewable generators to maximize their utilization.
- Positive weights for conventional generators (e.g., gas, oil), with higher values for more emission-intensive units.

Weighting methodology enables simultaneous optimization of:

1. Maximization of renewable energy penetration.
2. Minimization of emissions through preferential dispatch of lower-emission conventional generators.

### Generator constraints

Generators in the system are subject to operational constraints that ensure they function within their technical capabilities. These constraints include limits on power output and restrictions on how quickly their output can change.

#### Generator power limits

To ensure that each generator operates within its rated capacity and minimum stable generation level, its active power output is bounded. This is mathematically represented as:

2 $${\overline{P} }_{n,g,t}^{min}\cdot {P}_{n,g}^{nom}\le {p}_{n,g,t}\le {\overline{P} }_{n,g,t}^{max}\cdot {P}_{n,g}^{nom}$$

- ${p}_{n,g,t}$: Active power output of generator $g$ at bus $n$ at time $t$ (MW).
- ${P}_{n,g}^{nom}$: Nominal capacity of the generator $g$ at bus $n$ (MW).
- ${\overline{P} }_{n,g,t}^{min}$ and ${\overline{P} }_{n,g,t}^{max}$: Per-unit minimum and maximum operational limits of the generator $g$ at bus $n$ at time $t$ (MW).
  - For **conventional generators**: usually constant across time, defining stable operating ranges. It may also be time-varying representing fuel availability.
  - For **renewable generators**: Time-varying, reflecting resource availability (e.g., wind speed, solar irradiation).

#### Generator ramp rate constraints

In addition to power limits, generators are subject to ramp rate constraints, which restrict how quickly their power output can change over time. These constraints are particularly crucial for thermal generators, as they reflect the thermodynamic and mechanical limitations that prevent instantaneous adjustments in output. The ramp rate constraints are modeled as:

3 $${p}_{n,g,t+1}-{p}_{n,g,t}\le 60{R}_{n,g}^{+}$$

4 $${p}_{n,g,t+1}-{p}_{n,g,t}\ge -60{R}_{n,g}^{-}$$

- ${R}_{n,g}^{+}$, ${R}_{n,g}^{-}$: Maximum ramp-up and ramp down rates of generator $g$ at bus $n$ (MW/min)
- The factor 60 converts ramp rates from MW/min to MW/hour, assuming hourly time step

### Transmission line constraint

In DC power flow transmission lines are sufficiently represented by a reactance ${X}_{l(a,b)}$. In the presented model transmission lines are power flow is bounded by:

5 $$-{\overline{P} }_{l\left(a,b\right)}\cdot {P}_{l\left(a,b\right)}^{nom}\le {p}_{l\left(a,b\right),t}\le {\overline{P} }_{l\left(a,b\right)}\cdot {P}_{l\left(a,b\right)}^{nom}$$

- ${p}_{l\left(a,b\right),t}$: Power flow through line $l$ from bus $a$ to bus $b$ at time $t$ (MW).
- ${P}_{l\left(a,b\right)}^{nom}$: Nominal capacity of the line $l$ (MW).
- ${\overline{P} }_{l\left(a,b\right)}$: Per-unit safety limit of line $l$.

The safety limit ${\overline{P} }_{l\left(a,b\right)}$ often imposed on transmission lines as a standard practice in power system planning to maintain reliability and security, particularly under contingency scenarios like N-1 conditions.

### Energy link constraint

Unlike transmission lines, links are not characterized by reactance. Instead, they are actively controllable components capable of representing high-voltage direct current (HVDC) lines, transformers, and may be used to model an electrolyzer that interconnects between different types of buses, such as electrical and hydrogen buses. The energy link is characterized by unidirectional energy flow bounded by:

6 $$0\le {p}_{el\left(a,b\right),t}\le {P}_{el\left(a,b\right)}^{nom}$$

- ${p}_{el\left(a,b\right),t}$: Energy flow through link $el$ from bus $a$ to bus $b$ at time $t$ (MW)
- ${P}_{el\left(a,b\right)}^{nom}$: Nominal capacity of the link e $l$ (MW).

### Storage unit

Storage units (e.g., CAES, Battery) are components that store and release energy, typically electricity, with explicit charging ${p}_{n,s,t}^{chrg}$ and discharging ${p}_{n,s,t}^{dis}$ power variables. Storge units are directly connected to an electrical bus.

#### Charge and discharge limits

Storage units have limits on how much power they can charge or discharge at any given time. These limits are determined by:

7 $$0\le {p}_{n,s,t}^{chrg}\le {P}_{n,s}^{nom}$$

8 $$0\le {p}_{n,s,t}^{dis}\le {P}_{n,s}^{nom}$$

- ${p}_{n,s,t}^{chrg}$, ${p}_{n,s,t}^{dis}$: Charging and discharging power of storage $s$ at bus $n$ at time $t$ (MW).
- ${P}_{n,s}^{nom}$: Nominal power capacity of storage $s$ at bus $n$ (MW).

#### Energy storage capacity constraint

The amount of energy that can be stored in a storage unit is limited by the physical or operational limits of the storage system that could be expressed as:

9 $$0\le {e}_{n,s,t}\le {H}_{n,s}{P}_{n,s}^{nom}$$

- ${e}_{n,s,t}$: Energy stored in storage unit $s$ at bus $n$ at time $t$ (MWh).
- ${H}_{n,s}$: Maximum duration (hours) storage $s$ at bus $n$ can supply its nominal power.

#### Energy balance constraint

The energy level in a storage unit at any given time step is determined by its previous energy level, and the energy charged or discharged during the current time step, accounting for charging and discharging efficiencies. This relationship tracks the state of charge and is expressed by the following energy balance equation:

10 $${e}_{n,s,t}={e}_{n,s,t-1}+{\eta }_{n,s}^{chrg}\cdot {p}_{n,s,t}^{chrg}-{{\eta }_{n,s}^{dis}}^{-1}\cdot {p}_{n,s,t}^{dis}$$

- ${\eta }_{n,s}^{chrg}$, ${\eta }_{n,s}^{dis}$: Charging and discharging efficiencies ($\eta <1$).
- The term ${{\eta }_{n,s}^{dis}}^{-1}$ accounts for losses, requiring more energy withdrawal than delivered during discharge.

#### Cyclic operation constraint

This constraint enforces equivalent initial and terminal storage levels, preventing artificial depletion at the end of the optimization horizon and ensuring sustainability of operation.

11 $${e}_{n,s,1}={e}_{n,s,24}={E}_{n,s}^{init}$$

${E}_{n,s}^{init}$: Initial energy level (MWh).

### Energy store

Energy stores $es$ are reservoirs holding energy, potentially in non-electrical forms (e.g., hydrogen, heat). They often have a single net power variable ${p}_{n,es,t}$​ representing injection or withdrawal, typically connected to non-electrical buses or via energy links.

#### Energy store capacity constraint

Energy stores, such as hydrogen tanks or thermal storage, have a maximum capacity for holding energy. This limitation is respected by the model and is represented by:

12 $$0\le {e}_{n,es,t}\le {E}_{n,es}^{nom}$$

- ${e}_{n,es,t}$: Energy in store $es$ at bus $n$ at time $t$ (MWh).
- ${E}_{n,es}^{nom}$: Nominal capacity of store $es$, at bus $n$ at time $t$ (MWh).

#### Energy store balance constraint

The energy in the store changes based on the net input or output of energy over time. This is modeled by:

13 $${e}_{n,es,t+1}={e}_{n,es,t}+{p}_{n,es,t}$$

${p}_{n,es,t}$: Net energy input/output (MW), positive for input, negative for output.

#### Hydrogen stores constraint

For this stage of the research, the modeling framework considers all components designated as energy stores (es) to be hydrogen storage facilities. A key assumption is the initialization of these facilities to an empty state at the start of the simulation:

14 $${e}_{n,es,1}=0$$

These zero initial conditions imply that the final inventory level ${e}_{n,es,24}$ at the end of the optimization horizon (day) represents the net quantity of hydrogen generated and accumulated by the system. Thus, the minimum hydrogen production requirement:

15 $${\sum }_{n,es}\frac{{e}_{n,es,24}}{{E}^{{h}_{2},spec}}\ge {M}^{{h}_{2},min}$$

- ${E}^{{h}_{2},spec}$: Specific energy density of hydrogen (MWh/ton).
- ${M}^{{h}_{2},min}$: Minimum daily hydrogen production requirement (tons).
  16 $${e}_{n,es,t} \le {E}_{n,es}^{nom}$$

- ${E}_{n,es}^{nom}$: Maximum allowable daily hydrogen production (tons).

#### Total emissions constraints

This constraint imposes an upper bound on total system emissions, facilitating compliance with environmental targets and decarbonization objectives.

17 $${\sum }_{n,g,t}{p}_{n,g,t}\cdot {M}_{n,g}^{C{O}_{2}}\le {M}^{C{O}_{2},max}$$

where ${M}_{n,g}^{C{O}_{2}}$: Emission factor of generator $g$ at bus $n$ (MWh/ton). ${M}^{C{O}_{2},max}$: Maximum allowable daily emissions (tons).

### DC power flow

The DC power flow model linearizes AC equations, by assuming:

1. Voltage magnitudes are 1.0 p.u.
2. Small voltage angle differences: $sin({\theta }_{a} - {\theta }_{b}) \approx ({\theta }_{a} - {\theta }_{b})$.
3. Negligible line resistance: (${X}_{l}$ > > ${R}_{l}$).

#### Kirchhoff’s voltage law (KVL)

Under DC power flow assumptions, the relationship between the active power flow ${p}_{l\left(a,b\right),t}$ on a line from $l$ bus $a$ to bus $b$, with the line reactance ${X}_{l}$, and the bus voltage angles ${\theta }_{a}$ and ${\theta }_{b}$ simplifies to a linear equation:

18 $${p}_{l\left(a,b\right),t}\approx \frac{{v}_{a,t}{v}_{b,t}}{{X}_{l}}\text{sin}\left({\theta }_{a,t}-{\theta }_{b,t}\right)\approx \frac{{\theta }_{a,t}-{\theta }_{b,t}}{{X}_{l}}$$

Rearranging the power flow equation gives the voltage angle difference across a transmission line as a function of power flow:

19 $${\theta }_{a,t}-{\theta }_{b,t}={X}_{l}{p}_{l\left(a,b\right),t}$$

According to **Kirchhoff’s Voltage Law (KVL)**, the algebraic sum of voltage differences around any closed loop (or mesh) in a circuit must be zero. In the context of DC power systems, this implies that the sum of voltage angle differences across the lines forming a mesh must be zero:

20 $$\sum_{l(a,b)\in m}({\theta }_{a,t}-{\theta }_{b,t})=0$$

where the summation is taken over all lines $l\left(a,b\right)$ that form the mesh $m$, following the assigned direction of traversal. Substituting the expression ${X}_{l}{p}_{l\left(a,b\right),t}$ for the difference $\left({\theta }_{a,t}-{\theta }_{b,t}\right)$ yields:

21 $${\sum }_{l(a,b)\in m}{X}_{l\left(a,b\right)}{p}_{l\left(a,b\right),t}=0$$

#### Nodal power balance constraint

At each bus in the network, the net power injection must equal the power demand, accounting for generation, storage, energy links, and transmission flows. This fundamental principle of power conservation is enforced by the following constraint.

22 $$\begin{aligned} d_{{n,t}} & = \sum _{g} p_{{n,g,t}} + \sum _{s} \left( {p_{{n,s,t}}^{{dis}} - p_{{n,s,t}}^{{chrg}} } \right) - \sum _{{es}} p_{{n,es,t}} \\ & \quad + \sum _{{b = n}} p_{{l\left( {a,b} \right),t}} - \sum _{{a = n}} p_{{l\left( {a,b} \right),t}} - \sum _{{a = n}} p_{{el\left( {a,b} \right),t}} + \sum _{{b = n}} \eta _{{el\left( {a,b} \right)}} p_{{el\left( {a,b} \right),t}} \\ \end{aligned}$$

- ${d}_{n,t}$: Demand at bus $n$ at time $t$ (MW).
- $${\eta }_{el\left(a,b\right)}$$: Link $$el$$ efficiency ($$\eta <1$$).

Summations are over components connected to bus $n$: Generation, storage (discharge—charge), energy stores (consumption), transmission lines (in—out), and energy links (out—in, adjusted by efficiency ${\eta }_{el\left(a,b\right)}$). This fundamental constraint ensures power conservation at each bus $n$ at time $t$.

### Daily assessment cost structure

In this study, the overall cost framework is structured around **three primary daily economic components** that reflect the operational, environmental, and capital aspects of the energy system:

1. *Operational Cost of Electricity Generation*: This represents the **day-to-day running cost** of the power system. It is calculated as the **incremental cost of generation (in $/MWh) multiplied by the electrical output (in MWh)** of the conventional generators. This cost captures fuel expenses, variable operations and maintenance, and any other marginal cost associated with producing electricity.
2. *Social Carbon Credit Revenue*: In recognition of the environmental value of emissions reduction, the model includes **revenues from avoided CO**_2_** emissions**. These are computed by multiplying the **total tonnes of carbon emissions avoided** by the **social cost of carbon or carbon credit price (in $/tonne CO**_2_**)**. This component reflects the societal benefit of displacing fossil-fuel-based generation with cleaner energy sources or through system efficiency improvements.
3. *Equivalent Daily Cost of Storage*: The third component captures the capital and lifecycle cost of energy storage assets, expressed daily. This is based on the **Levelized Cost of Storage (LCOS)**, a comprehensive metric that includes all costs incurred over the lifetime of a storage system. LCOS accounts for capital expenditure (CAPEX), operations and maintenance (O&M), financing, and long-term expenses such as battery replacements or system augmentation. To translate LCOS into a daily cost, the system’s **energy rating (in MWh)** is multiplied by the LCOS (in $/MWh). For example, a 1,000 MWh storage facility with an LCOS of $110/MWh would incur an equivalent daily cost of **$110,000**. This value represents the average daily revenue the system must generate to recover its full lifetime cost and remain financially viable.

Together, these three components, represented in (23), form a balanced and integrated approach to cost assessment—one that considers not only the direct operational costs but also the environmental benefits and the long-term economic impact of energy storage technologies.

23 $$c={\sum }_{n,g,t}{C}_{n,g}{p}_{n,g,t}+{\sum }_{n,es}{C}_{n,es}{E}_{n,es}^{nom}-{C}^{C{O}_{2}}\left({M}^{C{O}_{2},init}-{\sum }_{n,g,t}{p}_{n,g,t}\cdot {M}_{n,g}^{C{O}_{2}}\right)$$

- $$c$$: Total daily system cost ($).
- $${C}_{n,g}$$: Incremental cost of generator $$g$$, at bus $$n$$ ($/MWh).
- $${C}_{n,es}$$: The daily storage levelized cost per MWh of storage capacity.
- $${C}^{C{O}_{2}}$$: Social cost of carbon ($/tCO_2_)
- $${M}^{C{O}_{2},init}$$: Initial daily emissions (tonne).

### Generating data for the testing model on IEEE 9 bus system

**Fig. 1:**
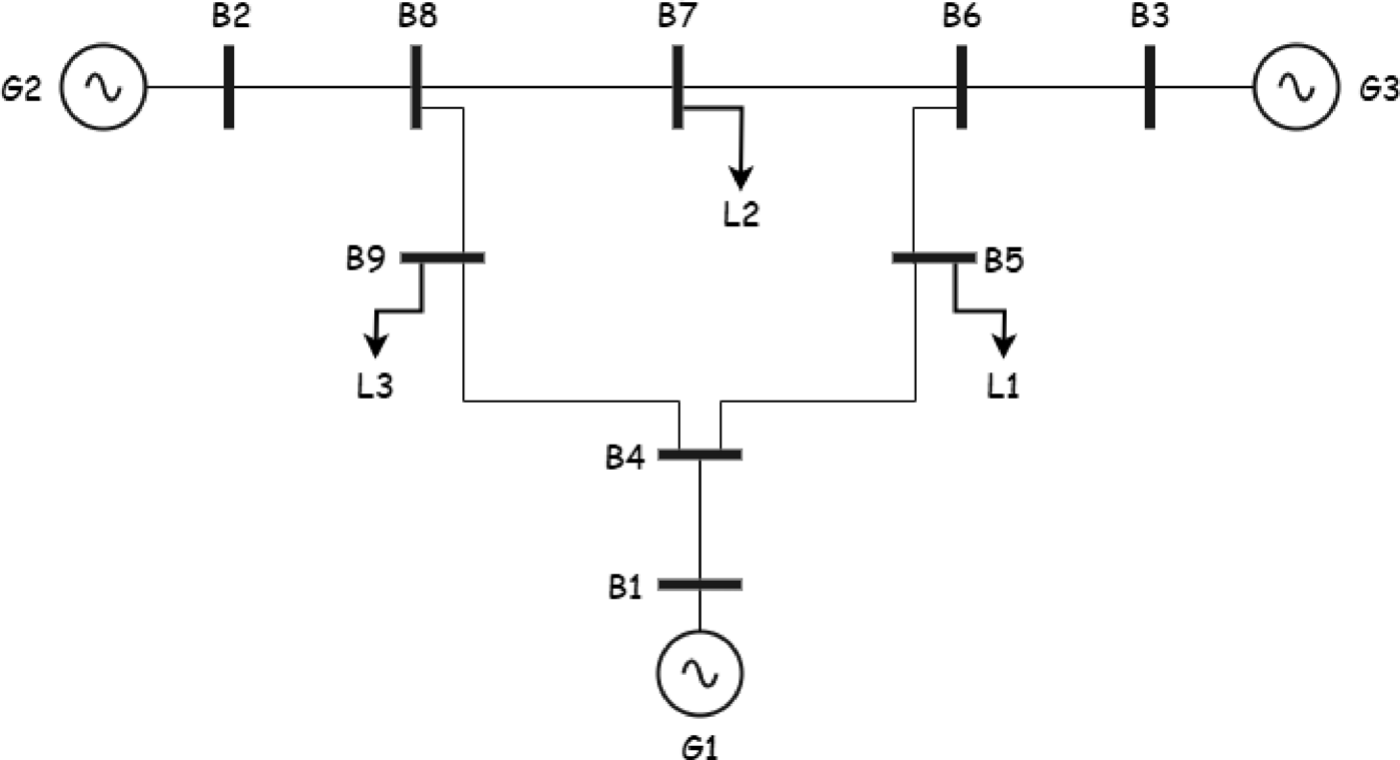
the IEEE case 9.

In this section realistic data is used to test the model and acquire important intuitions for The IEEE 9 bus system is shown in Fig. 1. The IEEE 9-bus system is a standard transmission system consists of 9 buses, 3 conventional generators and 3 loads^34^. This system is widely used in power system research due to its simplicity and well-documented characteristics. It provides a controlled environment to validate the proposed multi-objective optimization framework before scaling it to more complex, real-world systems like Oman’s power grid. While it does not fully replicate the specific topology or operational conditions of Oman’s network, its use ensures that the methodology can be rigorously tested and refined.

In the subsequent sections the data used in the case study and the modifications done on the system to integrate renewables, storage, and green hydrogen production are summarized.

### Generators data

Table 1 presents the operational parameters for three conventional power generators in the system. The Table specifies the maximum power output (P limits) for each generator, with capacities given in megawatts for generators G1, G2, and G3. Maximum ramp rates are expressed as percentages per minute, where gas-type generators G1 and G2 exhibit identical ramping capabilities, while the oil-type generator G3 demonstrates significantly higher ramping performance. The Table also includes incremental cost data according to reference^35^. Additionally, emissions characteristics are provided, indicating that gas generators produce lower carbon emissions per megawatt-hour compared to the oil generator, which exhibits a higher carbon footprint, with associated social costs of 50 $/tCO_2_ as reported in^36^.

**Table 1:** Convention generators data.

	P limits (MW)	Max ramp rate (% per min)	Type	Incremental cost ($/MWh)	Emissions rate (tco2/MWh)
G1	195	6	Gas	45	0.2
G2	240	6	Gas	40	0.2
G3	214	15	Oil	100	0.25

#### Loads profile

**Fig. 2:**
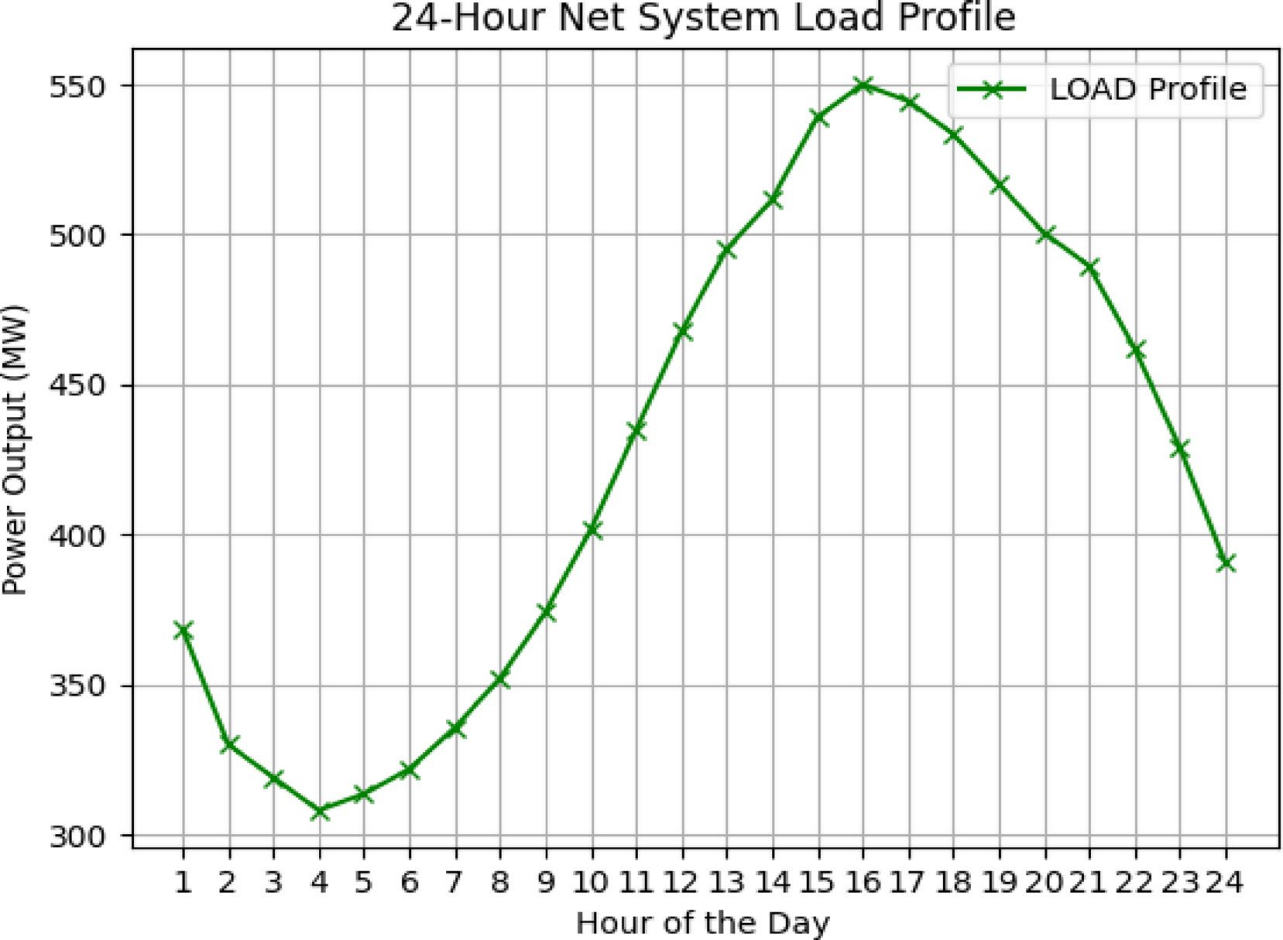
Total.

Fig. 2 illustrates the total system load, with all three loads following the same profile. The first load accounts for 28% of the total load and is located at bus 5, the second load constitutes 32% at bus 7, and the third load represents 40% at bus 9.

### Storage data

This study employs three types of energy storage systems to enhance renewable energy penetration levels. The storage systems are characterized by their nominal power, expressed as a percentage of renewable capacity, and their supply duration in hours, which represents the reservoir capacity for pumped hydro or compressed air energy storage (CAES) systems. For lithium-ion batteries, the supply duration is typically limited to 2 h to prevent rapid degradation. Table 2 presents the typical parameter values utilized in this study, including charging and discharging efficiencies. The table also includes incremental cost data according to reference^37^.

**Table 2:** Energy storage data.

	Pumped hydro (PH)	Battery	CAES
Supply duration (hour)	8h	2h	12h
Charging efficiency	0.87	0.95	0.85
Discharging efficiency	0.85	0.95	0.7
Average daily levelized cost	130 $/MWh	230 $/MWh	111 $/MWh

### Green hydrogen production

In this study, hydrogen generation is assumed to use an advanced alkaline electrolyzer with an efficiency (${\eta }_{el\left(a,b\right)}$) of 70%, a standard value for modern large-scale systems. The produced hydrogen is stored in a large-capacity, high-pressure gaseous systems because this method offers a balance between technical feasibility and economic viability. These high-pressure storages allow for rapid charging and discharging, making them suitable for applications that require dynamic operation, such as integrating intermittent renewable energy sources. Moreover, it avoids the complexities and energy losses associated with cryogenic or solid-state storage methods^38^. The storage system is assumed to maintain hydrogen at an energy density (${E}^{{h}_{2},spec}$) of $33.6 MWh/ton$, which corresponds to its lower heating value (LHV). The LHV represents the energy released during combustion without recovering heat from the resulting water vapor, a common assumption in energy systems where vapor condensation is impractical^39^. Throughout this study, hydrogen stores capacities ${E}_{n,es}^{nom}$ are assumed to be very high, almost unlimited compared to system size allowing all excess renewable energy to be converted to green hydrogen. This assumption enables an assessment of the maximum potential for hydrogen production, offering an upper bound for what could be achieved with sufficient infrastructure. In this study, the model is allowed to utilize all excess renewable energy for green hydrogen production. The minimum and maximum daily levels of hydrogen production are considered 3 tonnes and 50 tonnes respectively.

### Renewables profile

**Fig. 3:**
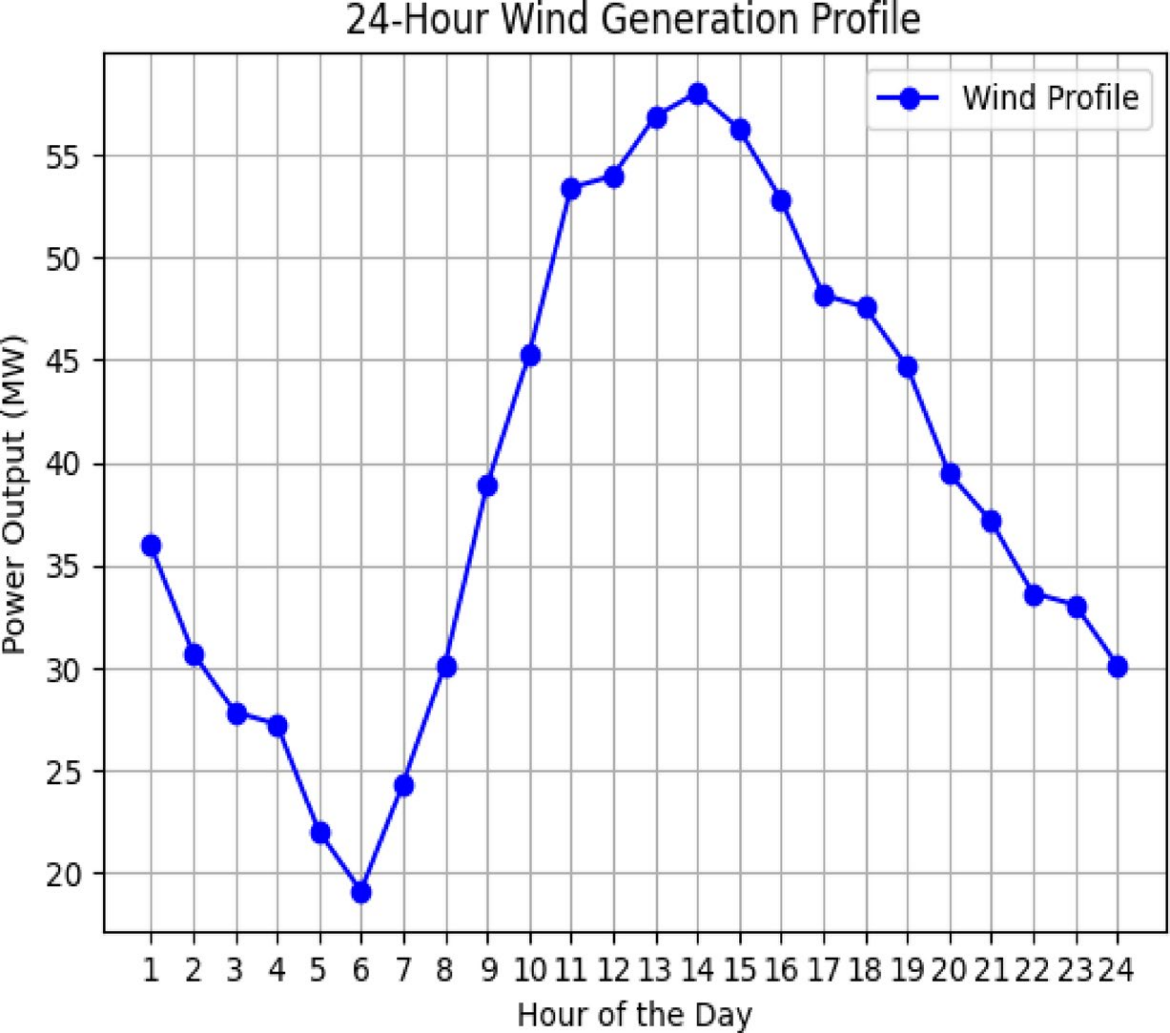
Wind profile.

**Fig. 4:**
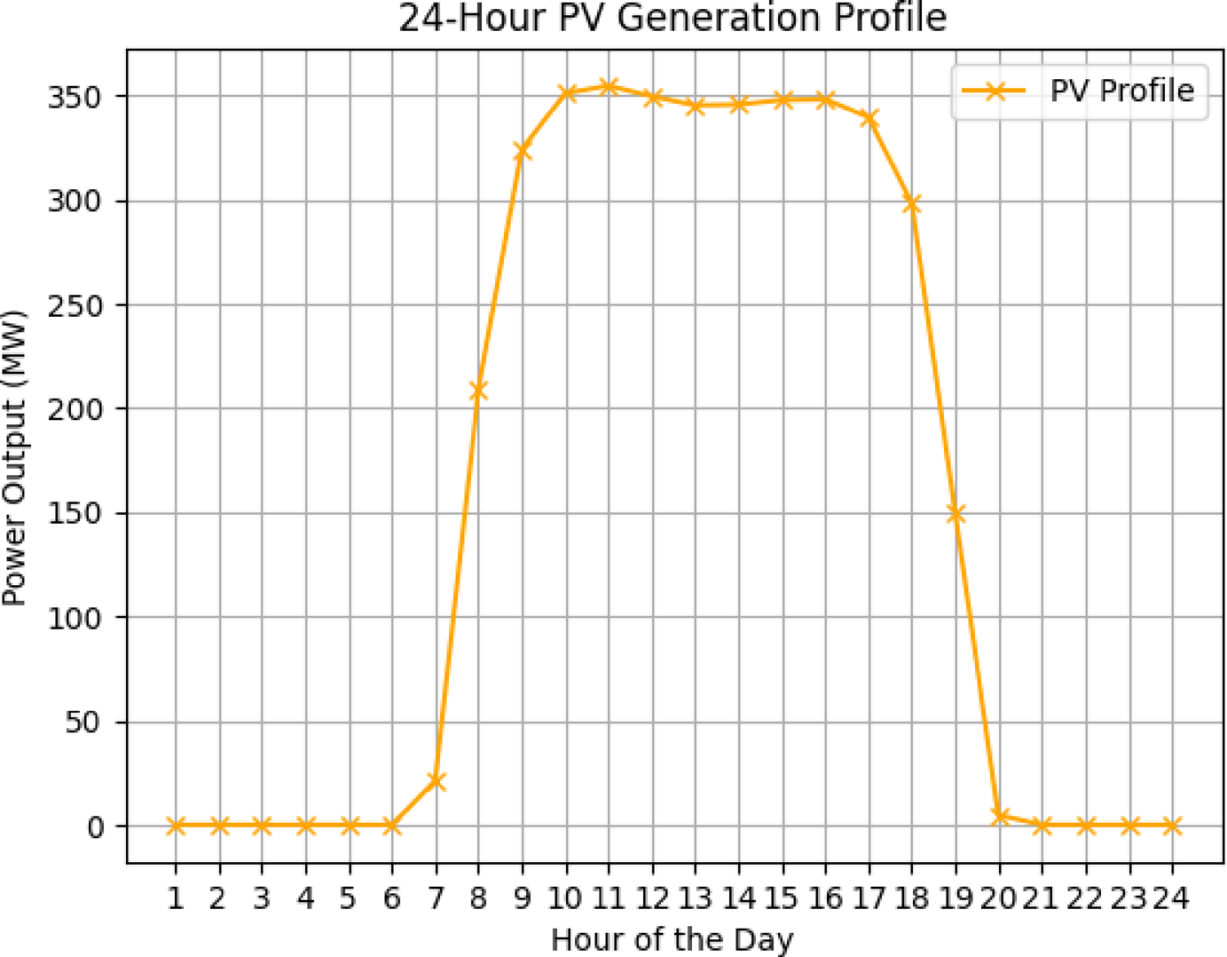
PV profile.

**Fig. 5:**
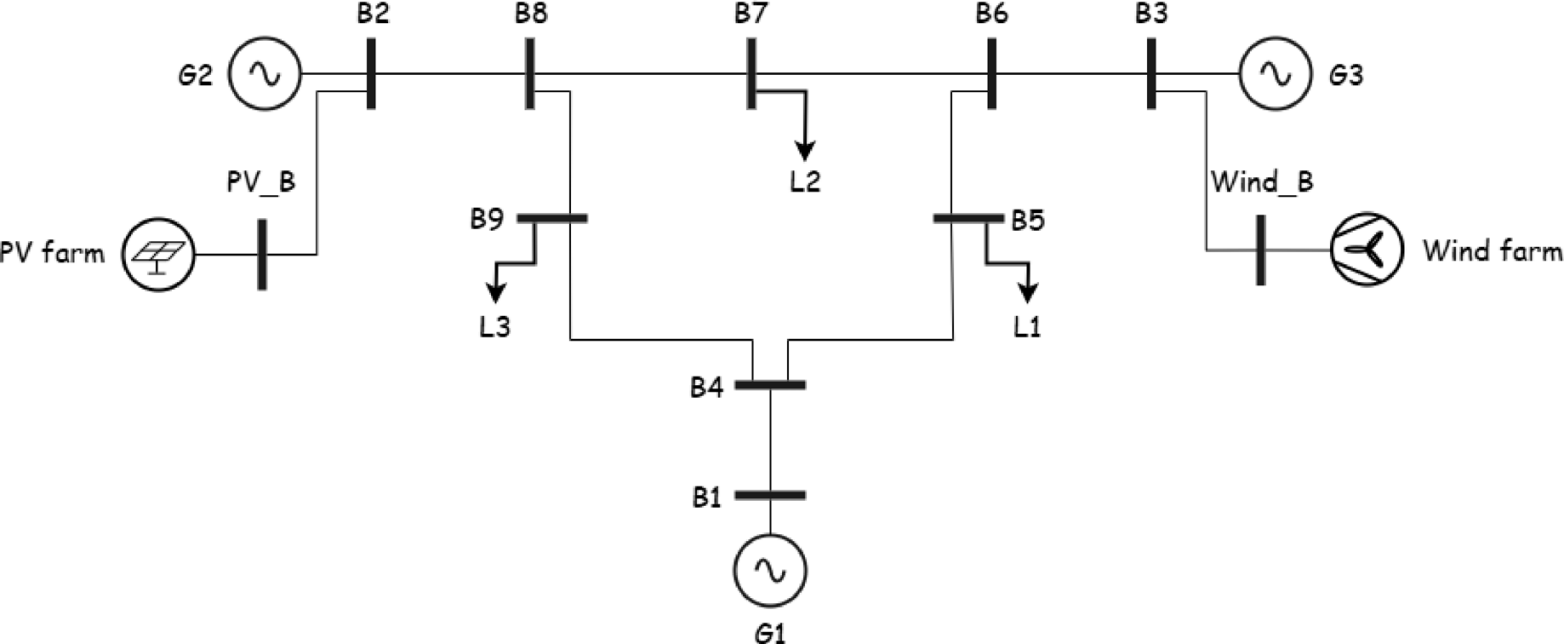
IEEE case 9 with PV and wind generation.

Realistic wind and PV profiles are used as shown in Figs. 3 and 4. The PV system with a rating of 360 MW is connected to bus 2, and the wind generation with a rating of 60 MW is connected to bus 3 respectively as shown in Fig. 5.

### Security constraint

The conservative threshold ${\overline{P} }_{l\left(a,b\right)}$ is adopted here as 0.7 due to the lack of specific operational data from Oman’s grid. While actual constraints may vary based on local standards, this threshold can be adopted to actual grid data ensuring a robust and secure grid operation.

## Results and discussion

Four cases will be examined in this section. The first case involves operating the system without any renewable energy sources or storage. The second case introduces renewable energy to evaluate the maximum achievable penetration level. In the third case, hydrogen production is studied under this penetration level. The fourth case considers the integration of various types of energy storage to support the renewable energy penetration and green hydrogen production.

### Case 1

System Configuration: The system operates solely with conventional generators (G1, G2, G3) on buses 5, 7, and 9, respectively, without renewable energy or storage. Line security constraints limit transmission flows to 70% of capacity. The system is operated without any renewable energy sources or storage as shown in Fig. 1.

The key findings:

- Renewable Penetration: 0% (no renewables integrated).
- Total Emissions: 2164.13 tCO_2_/day.
- Total Energy Demand: Approximately 10,287.75 MWh/day.
- Daily storge cost: 0.0 k$
- emissions revenue: 0.0 k$
- Operational costs: 676.1 k$
- Total cost: 676.1 k$

In this baseline scenario, the system relies entirely on conventional generation, producing 2164.13 tCO_2_/day. The total daily energy demand is approximately 10,287.75 MWh/day. To achieve 40% renewable penetration, renewable sources must supply at least 40% of that total energy demand.

### Case 2

*System Configuration*: Wind and PV generation are introduced on buses 2 and 3, respectively (Fig. 5), under transmission lines security constraints, without energy storage.

The key findings:

- Renewable Penetration: 28.65%.
- Renewable Energy Utilized: 2947.88 MWh/day
- Total Emissions: 1538.12 tCO_2_/day (28.92% reduction from baseline).

With renewable generation limited by grid constraints and no storage to shift excess energy, only 2976.88 MWh/day of the available 4778.64 MWh/day is integrated, achieving a maximum allowable penetration of 28.65%. This falls short of the 40% target (4155.02 MWh/day. Emissions decreased by 28.92%, reflecting partial displacement of conventional generation.

### Case 3

**Fig. 6:**
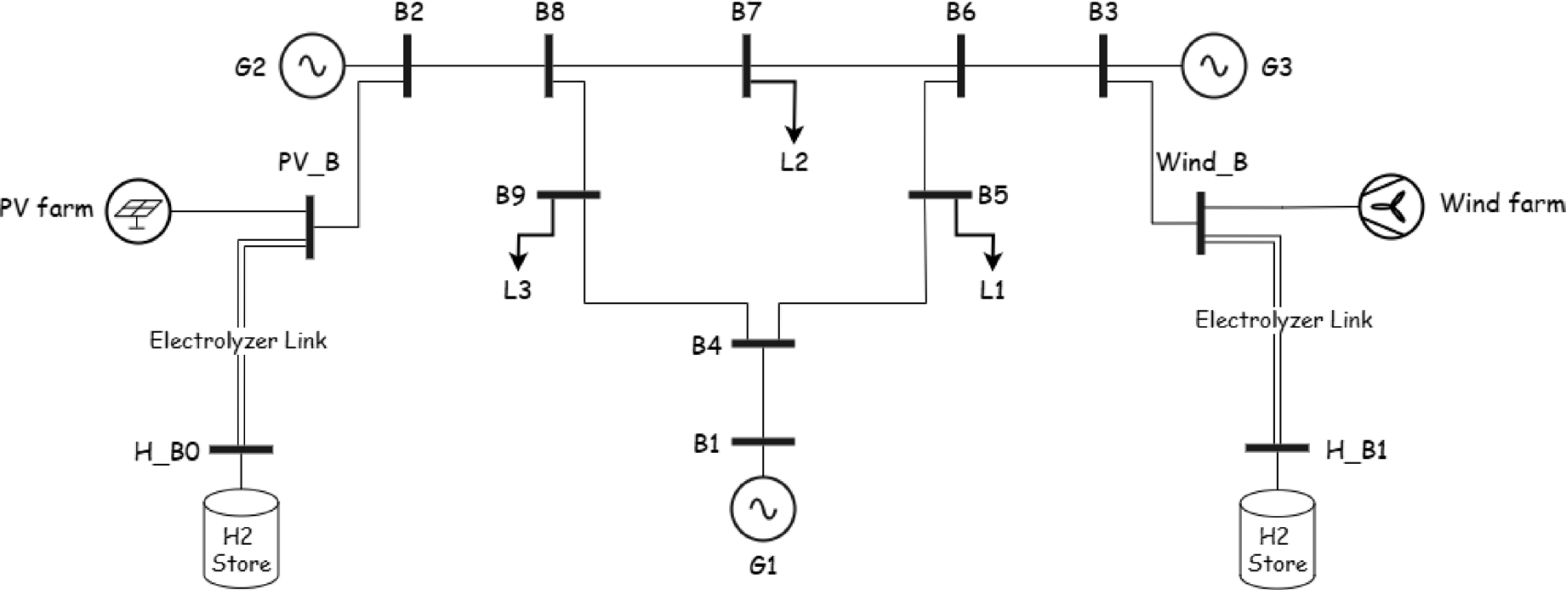
system under study in Case 3.

*System Configuration*: The system maintains 28.65% renewable penetration from Case 2, adding an electrolyzer link (efficiency 0.7) and a large-capacity hydrogen store (Fig. 6) to utilize excess renewable energy.

The key findings:

- Renewable Penetration: 28.65%.
- Renewable Energy Utilized for Electricity: 2947.88 MWh/day
- Green Hydrogen Production: 37 tons/day from excess renewable energy.
- Total Emissions: 1538 tCO_2_/day (28.92% reduction from baseline).

Here, the excess 1801.99 MWh/day of renewable energy, which was curtailed in Case 2, is directed to green hydrogen production, yielding 37 tons/day. Assuming a hydrogen energy density of approximately 33.6 MWh/ton, this corresponds to roughly 1247.57 MWh/day of green hydrogen energy output from the electrolyzer, adjusted for its 0.7 efficiency (1237.84/0.7 ≈ 1783.24 MWh/day of renewable energy). Penetration remains at 28.65%, as hydrogen production does not contribute to the electricity mix, but it enhances renewable utilization without increasing emissions beyond Case 2 levels.

### Case 4

**Fig. 7:**
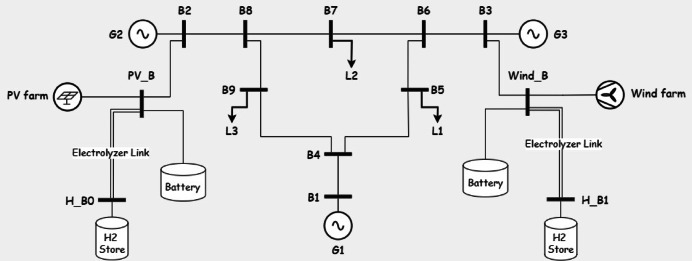
system under study in Case of using battery storage.

**Fig. 8:**
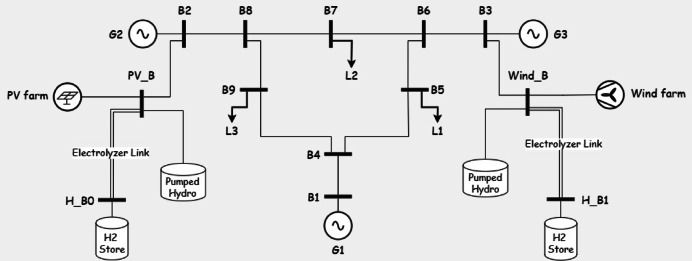
system under study in Case of using pumped hydro storage.

**Fig. 9:**
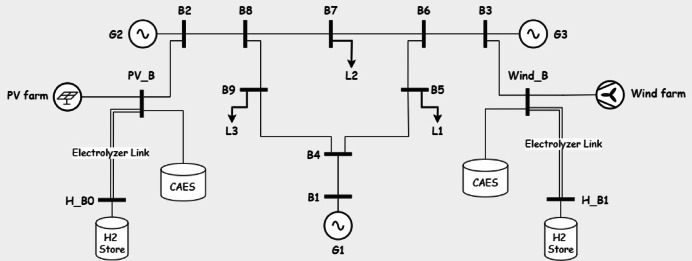
system under study in Case of using Compressed Air Energy Storage (CAES).

**Fig. 10:**
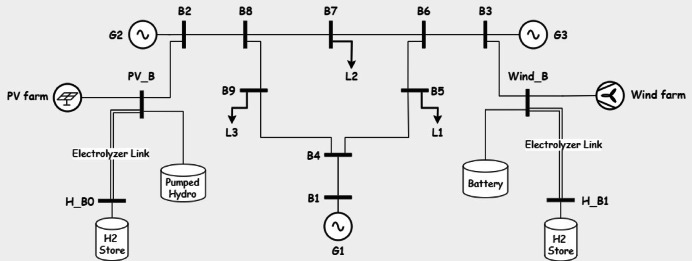
system under study in Case of using pumped hydro with batteries.

**Fig. 11:**
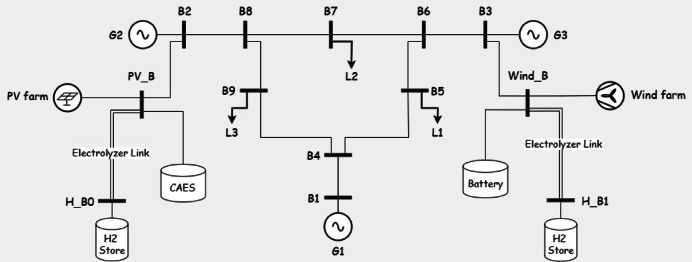
system under study in Case of using CAES with batteries.

*System Configuration*: Five storage types—Battery, Pumped Hydro (PH), Compressed Air Energy Storage (CAES), Pumped Hydro with Battery (PHB), and CAES with Battery (CAESB)—are integrated (Figs. 7, 8, 9, 10, 11). Storage capacity varies as a percentage of renewable capacity, with efficiencies and supply durations per Table 2.

**Fig. 12:**
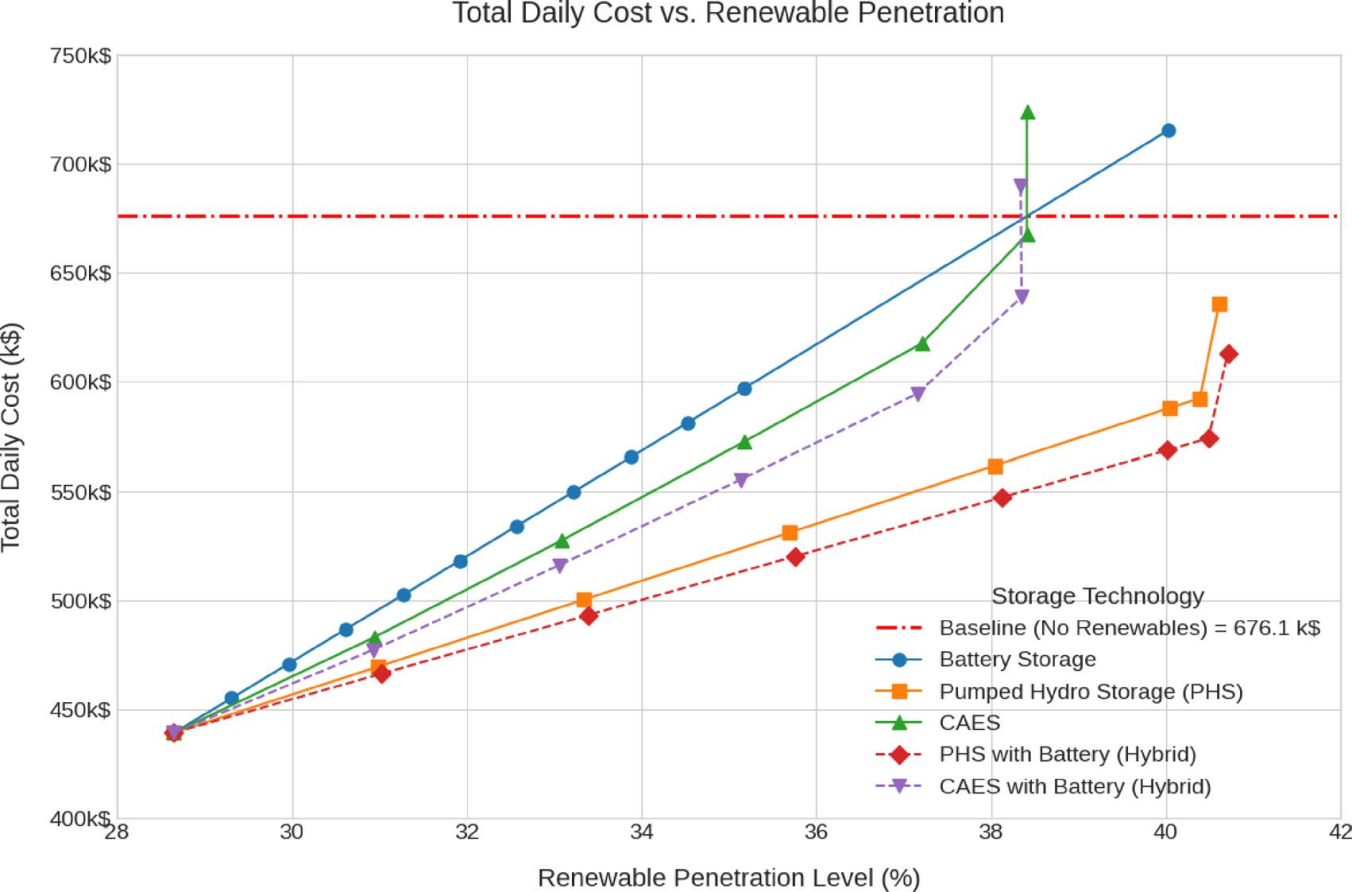
Total Daily cost versus Renewable Penetration for Battery, PH, CAES, Pumped hydro with battery (PHB), and Compressed air energy storage with battery (CAESB).

**Fig. 13:**
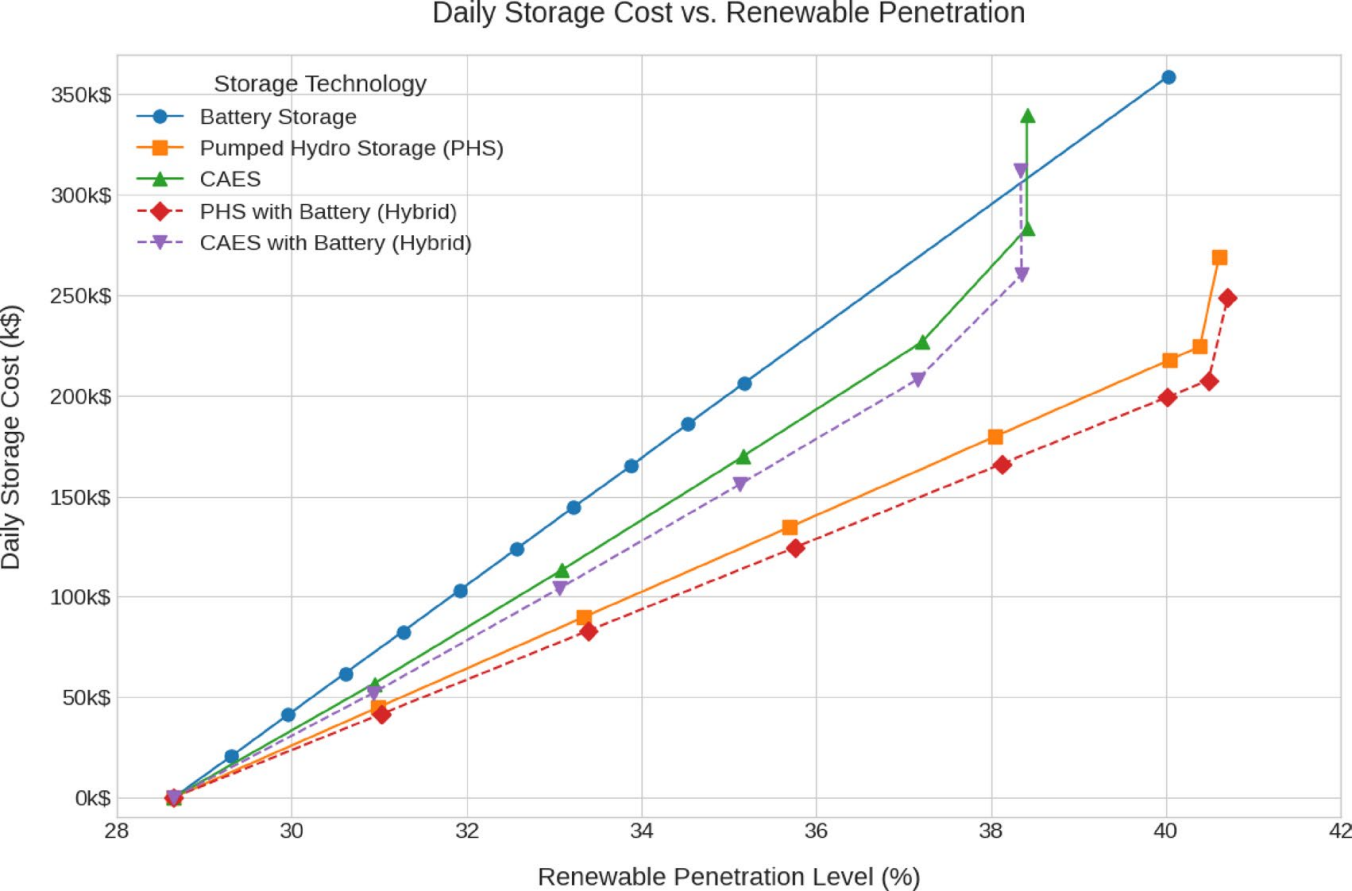
Dailt storage cost versus Renewable Penetration for Battery, PH, CAES, PHB, and CAESB.

**Fig. 14:**
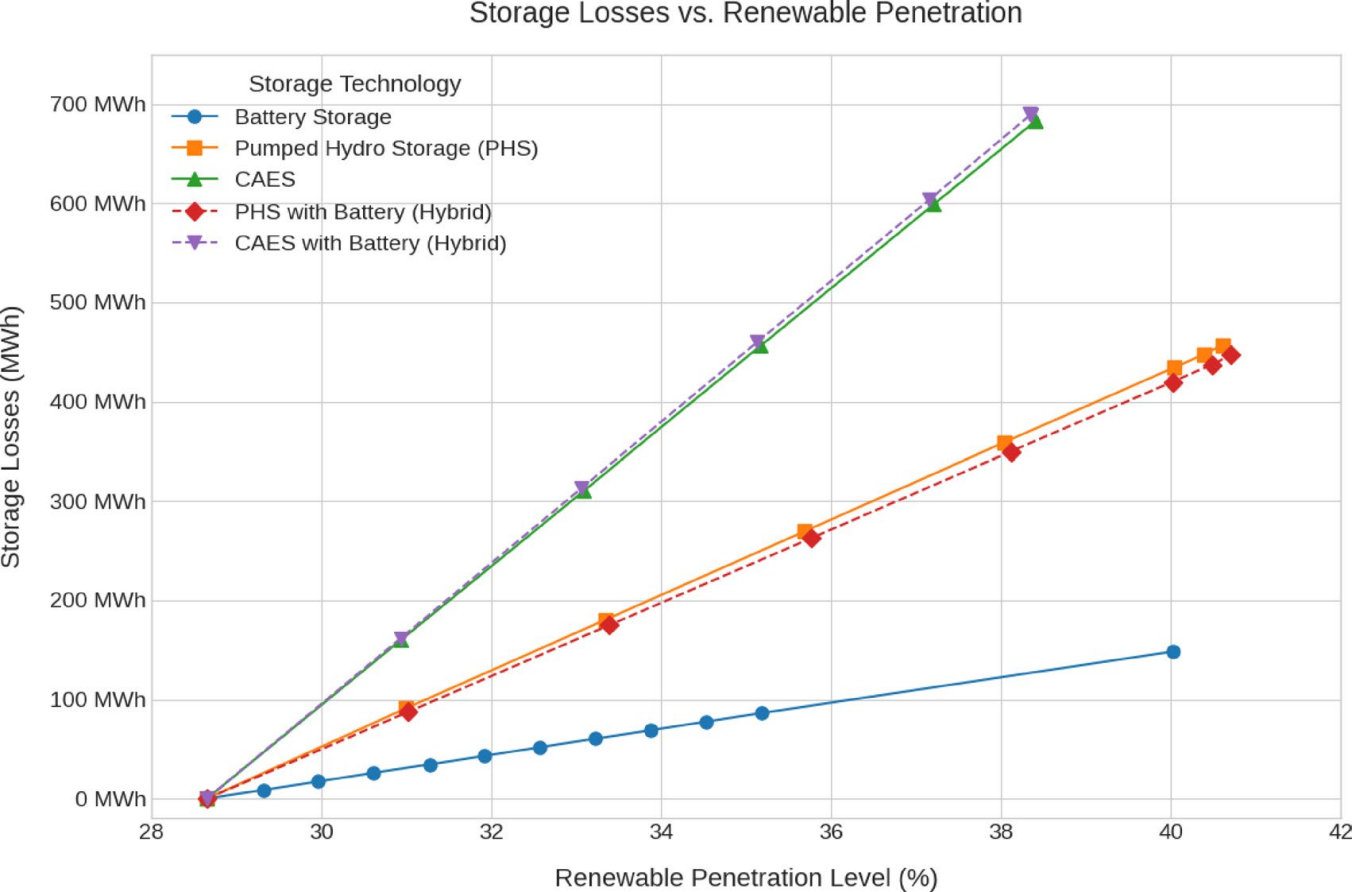
Storage Losses versus Renewable Penetration for Battery, PH, CAES, PHB, and CAESB.

**Fig. 15:**
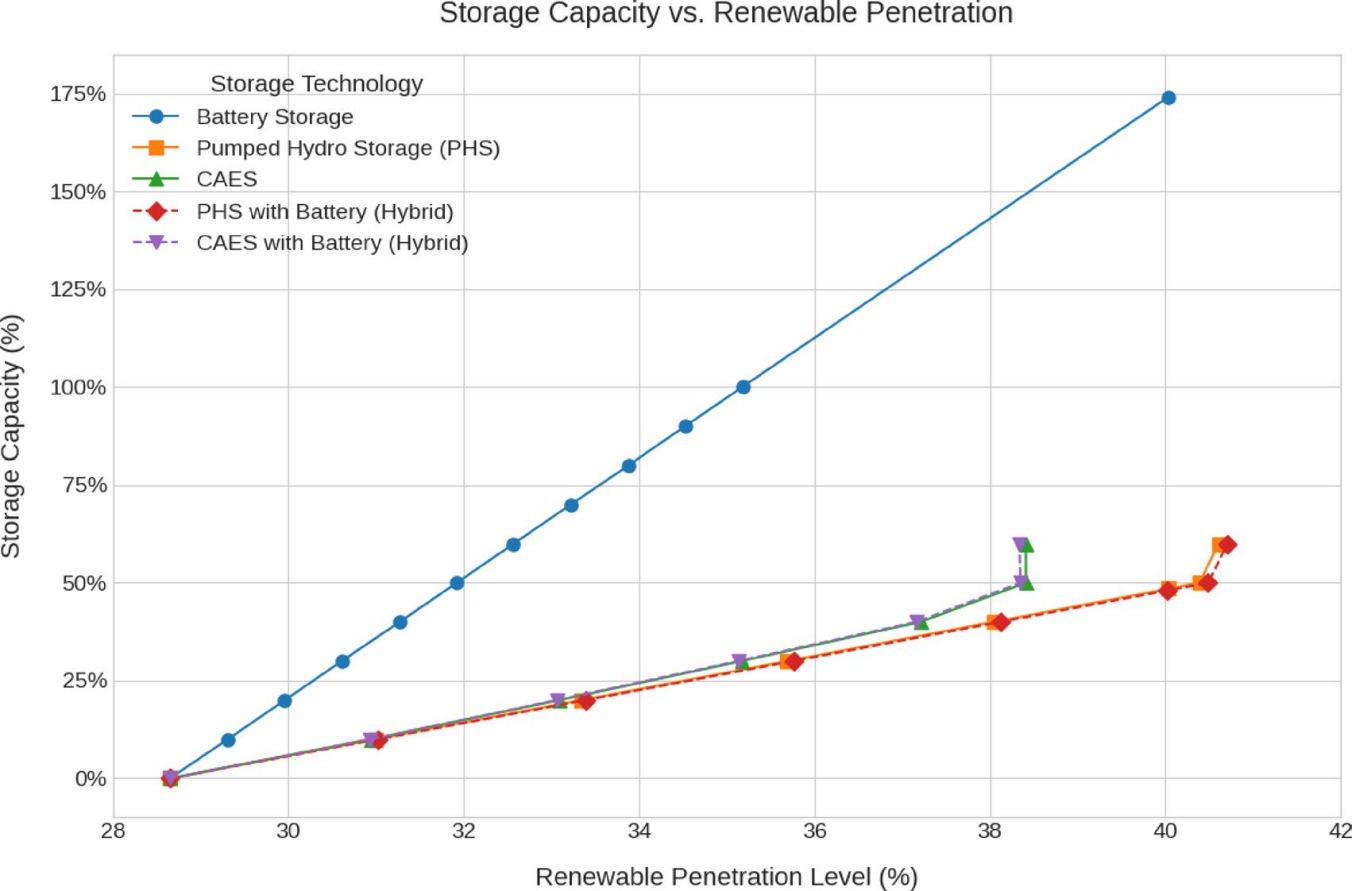
Storage Capacity versus Renewable Penetration for Battery, PH, CAES, PHB, and CAESB.

**Fig. 16:**
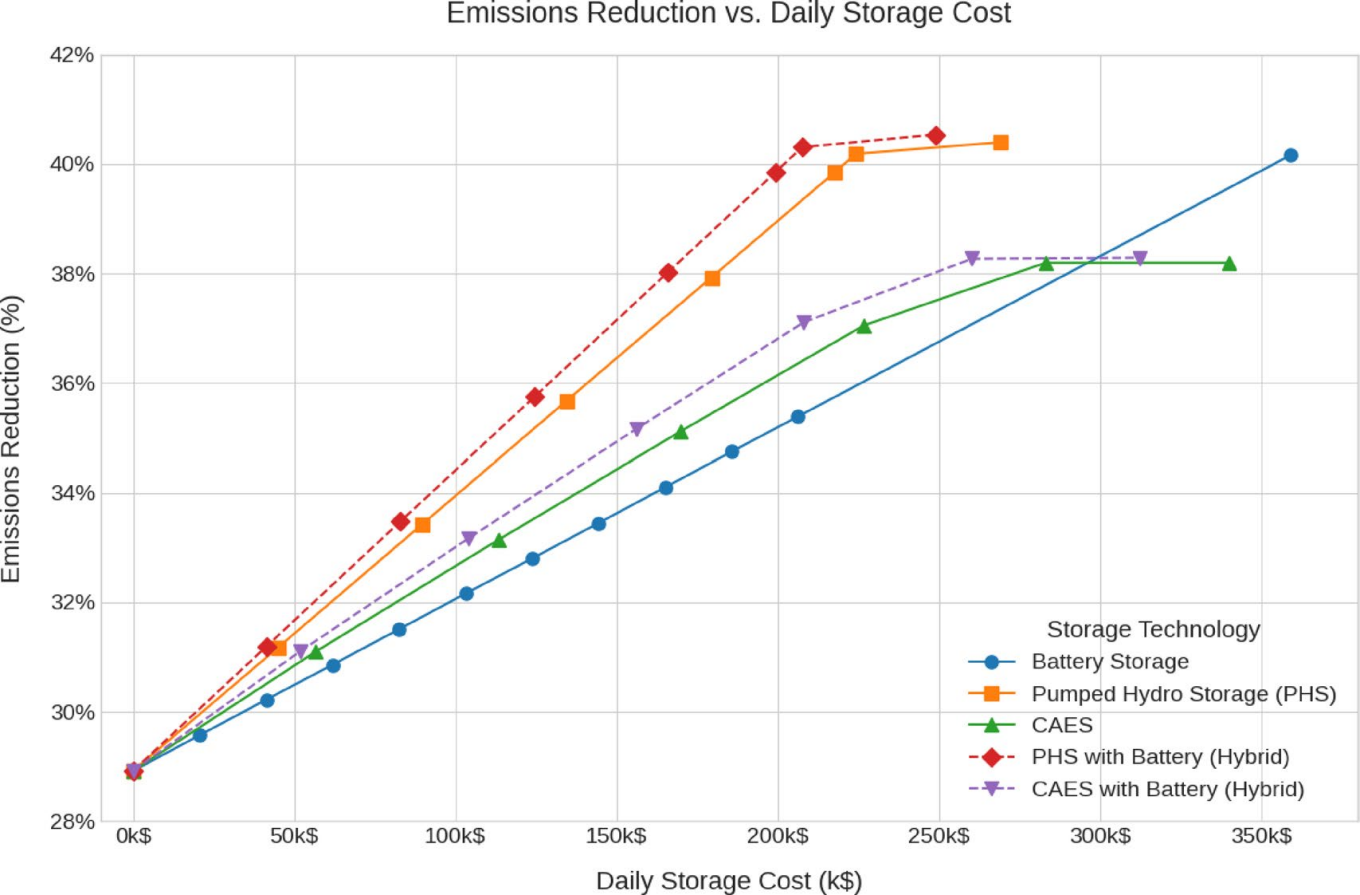
Emissions Reduction versus Daily storage cost for Battery, PH, CAES, PHB, and CAESB.

**Fig. 17:**
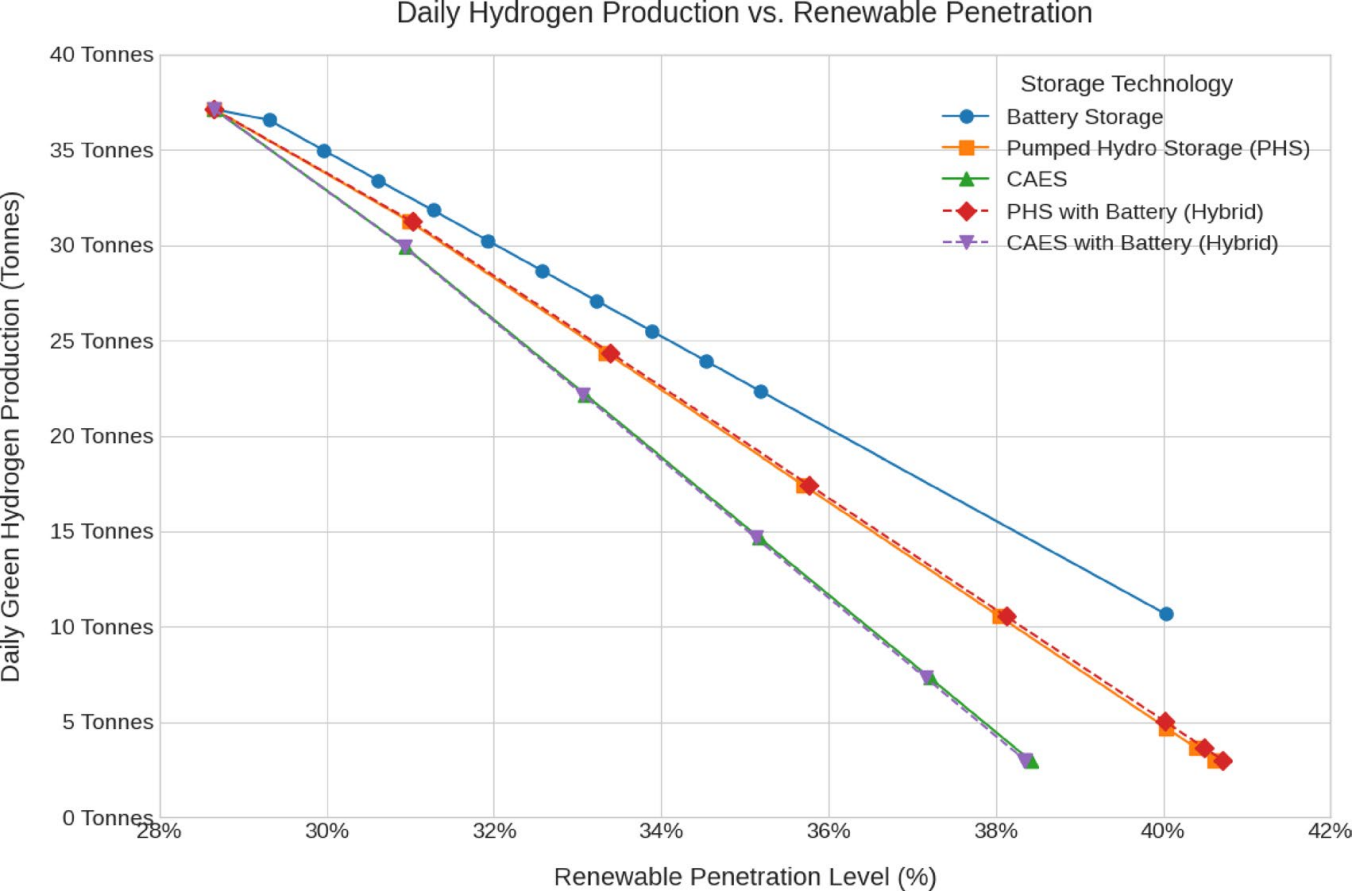
Daily Hydrogen Production versus Daily storage cost for Battery, PH, CAES, PHB, and CAESB.

The emissions, green hydrogen production, storage losses, renewables penetration and cost analysis for all scenarios are shown in Fig. 12, 13, 14, 15, 16 and 17.

Figures 12, 13, 14, 15, 16 and 17 show the following key findings:

At 40% renewable penetration, the total daily cost analysis (Fig. 12) reveals clear distinctions in the economic performance of various storage technologies. Among the options that reach 40%, Pumped Hydro with Battery (PHB) demonstrates the lowest total daily cost, approximately 570 k$/day, which is equivalent to around 84% of the baseline cost of 676.1 k$/day. This establishes PHB as the most cost-effective solution at this level of renewable integration. Pumped Hydro Storage (PH) follows with a total cost of around 590 k$/day, around 87% of the baseline. Compressed Air Energy Storage (CAES) caped at around 38% penetration level shows a higher cost of approximately 670 k$/day, and to achieve 40% penetration it needs extra investment in renewable energy. while Battery Storage reaches the highest cost, about 715 k$/day, equivalent to 105% of the baseline. On the other hand, hybrid CAES with Battery (CAESB) shows an improvement over CAES case but it failed again to reach 40% renewable penetration without more renewable investment. When comparing technologies at the same level of renewable integration, and considering only the least cost values for each, PHB stands out as the most economical option, achieving significant cost savings relative to both the baseline and other storage technologies.

In terms of Daily Storage Cost (Fig. 13), PHB again records the lowest value, around 200 k$/day, followed closely by PH at 220 k$/day. Battery shows significantly higher storage costs of approximately 360 k$/day. Similar to the previous metric, CAES and CAESB does not reach 40%, with moderate storage costs.

For Storage Losses (Fig. 14), Battery achieves the lowest losses, around 140 MWh. Among the technologies that do reach 40% penetration, PHB exhibits the next lowest losses at approximately 430 MWh, slightly outperforming PH, which records losses near 440 MWh. Both CAES and CAESB have the highest losses that lead to failure reaching the 40% penetration threshold at the current renewable investment.

In terms of Storage Capacity (Fig. 15), PH requires the lowest capacity at 40% penetration, approximately 49%, closely followed by PHB at 50%. Battery exhibits the highest demand, exceeding 175%, which represents a substantial increase in required capacity due to its limited 2 Hours supply duration. CAES and CAESB have slightly higher storage capacities than PH cases with maximum penetration level around 38%.

The Emissions Reduction versus Daily Storage Cost curve (Fig. 16) reveals that PHB and PH deliver the highest effective emissions reduction, approximately 40.5%, at a relatively low cost of 210 k$/day and 225 k$/day respectively. While Battery achieves 40%, but only at the significantly higher cost of 360 k$/day. CAES and CAESB saturated at 38% emissions reduction with moderate costs.

Finally, in Daily Hydrogen Production (Fig. 17), Battery leads with the highest output at 40% penetration, producing approximately 12 tonnes/day. PHB and PH follow with 5 tonnes/day and 4 tonnes/day, respectively. As with previous indicators, CAES and CAESB have the lowest hydrogen production profile without reaching the target penetration of 40%.

Based on the comparative data at 40% renewable penetration, PH with Battery (PHB) emerges as the most balanced and technically viable storage solution. It achieves the lowest total and storage costs, moderate storage losses, reasonable capacity requirements, and the highest emissions reduction, while still maintaining a practical level of hydrogen production. Although Battery storage excels in loss minimization and hydrogen output, its high cost and excessive capacity requirements undermine its practicality. CAES and CAESB, which consistently fail to operate at 40% penetration, clearly require additional renewable investment to become viable. Therefore, PHB offers the most efficient and sustainable storage configuration for supporting a 40% renewable energy system under current technical and economic constraints.

## Computational scalability

In this section, the scalability of the proposed optimization framework was assessed using the HiGHS solver on IEEE 30-, 39-, 57-, and 118-bus test systems, highlighting its computational efficiency and suitability for increasingly complex power system models. Running on Google Colab’s free tier—with 12.7 GB RAM, 107.7 GB disk space, and a Python 3 compute engine—the framework successfully completed all simulations with execution times ranging from 118.8 s (30-bus) to 561.5 s (118-bus). This performance demonstrates the framework’s robust scalability and practicality for real-world applications, even under constrained computing environments as shown in Table 3.

**Table 3:** Scalability.

IEEE system	IEEE 30	IEEE 39	IEEE 57	IEEE 118
Time(sec)	118.8	151.4	318.3	561.5

## Conclusion

The proposed framework successfully addresses existing gaps in renewable integration planning by incorporating detailed techno-economic analysis, emissions accounting, hydrogen production, and real-world operational constraints. Simulation results demonstrate that, among various storage strategies, the PHB configuration offers the most balanced performance, achieving 40% renewable penetration, reducing emissions by 40.5%, and achieving the lowest total cost at 570 k$/day. Battery storage, while effective in minimizing storage losses and maximizing hydrogen output (12 tonnes/day), proves economically less viable due to its high cost (715 k$/day) and capacity requirements. CAES and CAESB configurations fall short of the 40% penetration threshold, requiring further renewable investment. The framework’s scalability on IEEE test systems proves its computational robustness for application in large-scale grid planning scenarios.

Future research will focus on expanding the framework to include stochastic modeling of renewable variability, real-time grid operational dynamics, and demand-side management strategies. Incorporating detailed financial modeling, regulatory constraints, and policy incentives will enhance its practical applicability. Additionally, future studies will apply the model to a detailed Oman-specific network topology, enabling location-sensitive planning for storage and hydrogen infrastructure. Exploring the integration of second-life batteries, hybrid electrolyzer technologies, and sector coupling (e.g., water, transport) can further improve the robustness and sustainability of national energy transition strategies.

## Data Availability

The datasets used and/or analyzed during the current study are available from the corresponding author on reasonable request.

## Indices and sets

$t$: Set of hourly time steps (snapshots)
$n$: Set of buses (AC bus, DC bus, Hydrogen bus, etc.)
$g$: Set of generators (conventional or renewable)
*l*: Set of transmission lines
$s$: Set of storage units
$es$: Set of energy stores
$el$: Set of energy links
$m$: Set of meshes or cycles

## Variables

$$p_{n,g,t}$$: Active power output of generator $$g$$ at bus $$n$$ at time $$t$$ (MW)
$$p_{{l\left( {a,b} \right),t}}$$: Power flow through line $$l$$ from bus $$a$$ to bus $$b$$ at time $$t$$ (MW)
$$p_{{el\left( {a,b} \right),t}}$$: Energy flow through link $$el$$ from bus $$a$$ to bus $$b$$ at time $$t$$ (MW)
$$p_{n,s,t}^{chrg} ,p_{n,s,t}^{dis}$$: Charging and discharging power of storage $$s$$ at bus $$n$$ at time $$t$$ (MW)
$$e_{n,s,t}$$: Energy stored in storage unit $$s$$ on bus $$n$$ at time $$t$$ (MWh)
$$e_{n,es,t}$$: Energy in store $$es$$ at bus $$n$$ at time $$t$$ (MWh)
$$p_{n,es,t}$$: Net energy input/output (MW), positive for input, negative for output
$$\theta_{a} , \theta_{b}$$: Voltage angle at bus $$a and bus b$$
$$c$$: Total daily system cost ($)

## Parameters

$$P_{n,g}^{nom}$$: Nominal capacity of the generator $$g$$ at bus $$n$$ (MW)
$$\overline{P}_{n,g,t}^{min} ,\overline{P}_{n,g,t}^{max}$$: Minimum and maximum operational limits of generator $$g$$ at bus $$n$$
$$R_{{n,g}}^{ + } ,R_{{n,g}}^{ - }$$: Maximum ramp-up and ramp down rates of generator $$g$$ at bus $$n$$ (MW/min)
$$P_{{l\left( {a,b} \right)}}^{nom}$$: Nominal capacity of line $$l$$ (MW)
$$\overline{P}_{{l\left( {a,b} \right)}}$$: Per-unit safety limit of line $$l$$
$$P_{{el\left( {a,b} \right)}}^{nom}$$: Nominal capacity of link $$el$$ (MW)
$$P_{n,s}^{nom}$$: Nominal power capacity of storage $$s$$ at bus $$n$$ (MW)
$$H_{n,s}$$: Maximum duration (hours) storage $$s$$ at bus $$n$$ can supply its nominal power
$$\eta_{n,s}^{chrg} ,\eta_{n,s}^{dis}$$: Charging and discharging efficiencies ($$\eta < 1$$)
$$E_{n,s}^{init}$$: Initial energy level (MWh)
$$E_{n,es}^{nom}$$: Nominal capacity of store $$es$$, at bus $$n$$ at time $$t$$ (MWh)
$$E^{{h_{2} ,spec}}$$: Specific energy density of hydrogen (MWh/ton)
$$M^{{h_{2} ,min}}$$: Minimum daily hydrogen production (tons)
$$M^{{CO_{2} ,max}}$$: Maximum allowable daily emissions (tons)
$$x_{l}$$: Reactance of line $$l$$
$$d_{n,t}$$: Demand at bus $$n$$ at time $$t$$ (MW)
$$\eta_{{el\left( {a,b} \right)}}$$: Efficiency of energy link $$el$$
$$M_{n,g}^{{CO_{2} }}$$: Emission factor of generator $$g$$ at bus $$n$$ (MWh/ton)
$$W_{n,g}$$: Preference weight of generator $$g$$ at bus $$n$$
$$C_{n,g}$$: Incremental cost of generator g, at bus n ($/MWh)
$$C_{n,es}$$: The daily storage levelized cost per MWh of storage capacity
$$C^{{CO_{2} }}$$: Social cost of carbon ($/tCO2)
$$M^{{CO_{2} ,init}}$$: Initial daily emissions (tonne)
$$E_{n,es}^{nom}$$: Maximum allowable daily hydrogen production (tons)
